# Effect of Exercise Training on Bone Mineral Density in Post-menopausal Women: A Systematic Review and Meta-Analysis of Intervention Studies

**DOI:** 10.3389/fphys.2020.00652

**Published:** 2020-06-23

**Authors:** Mahdieh Shojaa, Simon Von Stengel, Daniel Schoene, Matthias Kohl, Giuseppe Barone, Laura Bragonzoni, Laura Dallolio, Sofia Marini, Marie H. Murphy, Aoife Stephenson, Minna Mänty, Mikko Julin, Tapani Risto, Wolfgang Kemmler

**Affiliations:** ^1^Institute of Medical Physics, Friedrich-Alexander University of Erlangen-Nürnberg, Erlangen, Germany; ^2^Department of Medical and Life Sciences, Institute of Precision Medicine, Furtwangen University, Furtwangen im Schwarzwald, Germany; ^3^Department for Life Quality Studies, University of Bologna, Bologna, Italy; ^4^Department of Biomedical and Neuromotor Science, University of Bologna, Bologna, Italy; ^5^School of Sport, Faculty of Life and Health Sciences, Ulster University, Coleraine, United Kingdom; ^6^Department of Public Health, University of Helsinki, Helsinki, Finland; ^7^Department of Strategy and Research, Vantaa, Finland; ^8^Department of Physiotherapy, Laurea University of Applied Sciences, Espoo, Finland

**Keywords:** exercise, training, bone mineral density, BMD, post-menopausal women

## Abstract

Osteoporosis is a major health problem in post-menopausal women (PMW). Exercise training is considered a cost-effective strategy to prevent osteoporosis in middle aged-older people. The purpose of this study is to summarize the effect of exercise on BMD among PMW. A comprehensive search of electronic databases was conducted through PubMed, Scopus, Web of Science, Cochrane, Science Direct, Eric, ProQuest, and Primo. BMD changes (standardized mean differences: SMD) of the lumbar spine (LS) femoral neck (FN) and/or total hip were considered as outcome measures. After subgroup categorization, statistical methods were used to combine data and compare subgroups. Seventy-five studies were included. The pooled number of participants was 5,300 (intervention group: *n* = 2,901, control group: *n* = 2,399). The pooled estimate of random effect analysis was SMD = 0.37, 95%-CI: 0.25–0.50, SMD = 0.33, 95%-CI: 0.23–0.43, and SMD = 0.40, 95%-CI: 0.28–0.51 for LS, FN, and total Hip-BMD, respectively. In the present meta-analysis, there was a significant (*p* < 0.001), but rather low effect (SMD = 0.33–0.40) of exercise on BMD at LS and proximal femur. A large variation among the single study findings was observed, with highly effective studies but also studies that trigger significant negative results. These findings can be largely attributed to differences among the exercise protocols of the studies. Findings suggest that the true effect of exercise on BMD is diluted by a considerable amount of studies with inadequate exercise protocols.

## Introduction

Osteoporosis is a disease characterized by low bone mass, microarchitectural deterioration of bone tissue, leading to enhanced bone fragility, and a consequent increase in fracture risk (1991). The disease is an important global public health problem (Compston et al., 2019). Due to the menopausal transition, and the corresponding decline of estrogen, post-menopausal women (PMW) in particular, are at high risk of osteoporosis (Christenson et al., 2012). Exercise training is considered to be a low cost and safe non-pharmaceutical treatment strategy for the protection of musculoskeletal health and fracture prevention (Kemmler et al., 2015; Beck et al., 2017; Daly et al., 2019), thus, many studies have focused on the effects of exercise on bone mineral density (BMD) in PMW (Bonaiuti et al., 2002; Howe et al., 2011; Marques et al., 2011a; Zhao et al., 2017). However, their effects on BMD, as the most frequently assessed parameter for bone strength, vary widely. Some studies even report a negative effect (vs. control) on BMD (Bassey and Ramsdale, 1995; Nichols et al., 1995; Choquette et al., 2011). Considering the large variety of intervention protocols that can be created when combining different types of exercise, exercise-parameters, and training-principles, there is no doubt that some loading protocols demonstrate favorable, while others trigger negative effects, on BMD. Additionally, participant characteristics vary considerably for parameters (e.g., menopausal status, bone status, training status) that might modulate the effect of exercise on BMD and thus may contribute to the low effect size of exercise reported by most meta-analyses (Kelley, 1998a,b; Martyn-St James and Carroll, 2011; Marques et al., 2011a; Zhao et al., 2017).

In the present systematic review and meta-analysis, we aimed to; (1) quantify the general effect of exercise on BMD at lumbar spine (LS) and proximal femur (PF) regions of interest (ROI) by meta-analytic techniques, (2) identify participants and exercise characteristics that explain the effect of exercise on BMD and (3) propose exercise recommendations to favorably affect BMD at the LS, femoral neck (FN) and total hip (tHip) ROI in PMW.

## Materials and Methods

### Literature Search

This review and meta-analysis follows the Preferred Reporting Items for Systematic Reviews and Meta-Analyses (PRISMA) statement (Moher et al., 2015) and was registered in advance in the International prospective register of systematic reviews (PROSPERO) (ID: CRD42018095097). A comprehensive search of electronic databases was conducted through PubMed, Scopus, Web of Science, Cochrane, Science Direct, Eric, ProQuest, and Primo for all articles published up to March 01, 2019, with no language restrictions. The search strategy utilized the population, intervention and outcome approach. The literature search was constructed around search terms for “bone mineral density,” exercise,” and “post-menopausal.”

A standard protocol for this search was developed and controlled vocabulary (Mesh term for MEDLINE) was used. Key words and their synonymous were used by applying the following queries, (“Bone” or “Bone mass” or “Bone status” or “Bone structure” or “Bone turnover” or “Bone metabolism” or “Bone mineral content” or “Skeleton” or “Bone Mineral Density” or “BMD” or “Bone Density” or “Osteoporoses” or “Osteoporosis” or “Osteopenia”) AND (“Postmenopause” or “Post-Menopause” or “Post-menopausal”) AND (“Exercise” or “Training” or “Athletic” or “Sport” or” “physical activity”) AND (“Clinical trial” or “Randomized clinical trial”). Furthermore, reference lists of the included articles were searched manually to locate additional relevant studies. Unpublished reports or articles for which only abstracts were available were not considered. Duplicate publications were identified by comparing author names, treatment comparisons, publication dates, sample sizes, intervention, and outcomes. In the case of unclear eligibility criteria or when the confirmation of any data or additional information was needed, the authors were contacted by e-mail.

### Inclusion and Exclusion Criteria

Studies were included if they met the following criteria: (a) randomized or non-randomized controlled trials with at least one exercise group as an intervention vs. one control group with habitual (sedentary) lifestyle or sham exercises; (b) participants were post-menopausal at study onset; (c) the training program lasted a minimum of 6 months; (d) BMD of the LS or/and the proximal femur regions “total hip” and/or “FN” were used as outcome measures; (e) baseline and final BMD assessment reported at least for one desired regions; (f) BMD measurement assessed by dual-energy X-ray absorptiometry (DXA) or dual-photon absorptiometry (DPA); (g) studies with ≤ 10% of participants on hormone replacement therapy (HRT), hormone therapy (HT), adjuvant endocrine therapy, antiresorptive, or osteoanabolic pharmaceutic agents (e.g., Bisphosphonate, Denosumab, Strontiumranelate) or drugs with a dedicated osteo-catabolic effect on bone metabolism, (glucocorticoids), albeit only if the number of users was similar between exercise and control.

Studies addressing (a) interventions applying novel exercise technologies (e.g., whole-body vibration) (b) mixed gender or mixed pre- and post-menopausal cohorts without separate BMD analysis for PMW; (c) PMW under chemo- and/or radiotherapy; (d) PMW with diseases that affect bone metabolism; (e) the synergistic/additive effect of exercise and pharmaceutic therapy, or (f) duplicate studies or preliminary data from the subsequently published study and review articles, case reports, editorials, conference abstracts, and letters were excluded from the analysis.

### Data Extraction

Titles and abstracts were screened by an independent reviewer (MS) to exclude irrelevant studies. Two reviewers (SV and MS) separately and independently evaluated full-text articles and extracted data from the included studies. Disagreement was resolved by discussion between the two reviewers; if they could not reach a consensus a third reviewer was consulted (WK). An extraction form was designed to record the relevant data regarding publication details (i.e., the first author's name, title, country and publication year), details of the study (i.e., design, objectives, sample size for each group), participants' characteristics (i.e., age, weight, BMI, years since menopause), description of intervention (i.e., type of exercise, intervention period, frequency, intensity, duration, sets and repetition), compliance (including number of withdrawals), risk assessment, BMD assessment tool and evaluated region, BMD values at baseline and study completion.

### Outcome Measures

Outcomes of interest were BMD at the LS and the proximal femur (FN and/or tHip) as assessed by Dual Energy X-Ray Absorptiometry (DXA) or Dual Photon Absorptiometry (DPA) at least at baseline and study end.

### Quality Assessment

Included articles were independently assessed for risk of bias using the Physiotherapy Evidence Database (PEDro) scale risk of bias tool (Sherrington et al., 2000; de Morton, 2009). This was completed by two reviewers from Germany (MS, SvS). Partners from Finland (MM, MJ, TR), Italy (LB, LD, SM, GB) or Northern Ireland (MHM, AS) acted as a third reviewer. Potential biases in studies were selection bias, performance bias, detection bias, attrition bias, and reporting bias using 11 criteria, however, the scale scores 10 items. The categories assessed were randomization, allocation concealment, similarity at baseline, blinding of participants and staff, assessor blinding, incomplete outcome data, intention-to-treat analysis, between groups comparison, and measure of variability. Scores ranged from 0 to 10 and points were awarded when a criterion was clearly explained; otherwise, a point was not awarded. Discrepancies were discussed with a review author from Germany (WK) until a consensus was reached. The methodological quality of the included studies was classified as follows: ≥7, high; 5–6, moderate; <5, low (Ribeiro de Avila et al., 2018).

### Data Synthesis

For sub-analyses, the intervention period was stratified as ≤ 8, 9–18, and >18 months by considering the remodeling cycle for cancellous and cortical bone (Eriksen, 2010). Post-menopausal status was categorized as early (≤ 8 years) and late (>9 years) (Harlow et al., 2012). We also classified the type of exercise into seven sub-groups including weight-bearing aerobic exercise (WB-AE), dynamic resistance training (DRT), Jumping+[resistance training (RT) and/or WB], WB+RT, Jumping, non-WB+RT and Tai Chi. Type of mechanical forces was categorized as joint reaction force (JRF), ground reaction force (GRF), and mix of JRF+GRF (Daly et al., 2019; Kemmler and von Stengel, 2019).

If the studies presented a confidence interval (CI) or standard errors (SE), they were converted to standard deviation (SD) by using standardized formulae (Higgins and Green, 2008). Where standard deviation was not given, authors were contacted to provide the missing data. When no reply was received or data were not available, the exact *p*-value of the absolute change of BMD was obtained to compute the SD of the change. In the case of unreported *p*-value, we calculated the SDs using pre and post SDs, and correlation coefficients with the following formula:

$$\begin{array}{l} \surd \text{S}{\text{D}}_{\text{pre}}^{2}+\text{S}{\text{D}}_{\text{post}}^{2}-\left(2\times \text{corr}\times \text{S}{\text{D}}_{\text{pre}}\times \text{S}{\text{D}}_{\text{post}}\right), \end{array}$$

where “*corr”* is the correlation coefficient which was imputed using the mean of the correlations available for some included studies. SD_pre_ and SD_post_ are the baseline and final standard deviation, respectively (Higgins and Green, 2008). This resulted in using a within-participant correlation of *r* = 0.95 and *r* = 0.94 in exercise and control groups at LS, respectively. At FN, the mean correlation was computed *r* = 0.82 among exercise groups and *r* = 0.85 for control groups. Finally, at the total hip, *r* = 0.97 and *r* = 0.98 were considered for intervention and control groups, respectively. When the absolute mean difference was not available, it was imputed by calculation of the difference between post- and pre-intervention. For those studies which measured BMD at multiple times, only the baseline and final values were included in the analysis.

### Statistical Analysis

The meta-analyses were performed using the package metaphor in the statistical software R (R Development Core Team, 2019). Effect size (ES) values were considered as the standardized mean differences (SMDs) combined with the 95% confidence interval (CI).

Random-effects meta-analysis was conducted by using the meta for package (Viechtbauer, 2010). Heterogeneity for between-study variability was implemented using the Cochran Q test and considered statistically significant if *p*-value < 0.05. The extent of heterogeneity was examined with the *I*^2^ statistics. *I*^2^ 0 to 40% is considered as low heterogeneity, 30 to 60%, and 50 to 90% represent moderate and substantial heterogeneity, respectively (Higgins and Green, 2008). For those studies with two different intervention groups, the control group was split into 2 smaller groups for comparison against each intervention group (Higgins and Green, 2008).

To explore potential publication biases, a funnel plot with regression test and the rank correlation between effect estimates and their standard errors (SEs), using the *t*-test and Kendall's τ statistic were conducted, respectively. The *p*-value < 0.05 was defined as the significant level for all tests.

Subgroup analyses were performed for menopausal status, intervention duration, type of exercise, and type of mechanical forces. Sensitivity analysis was conducted to try different values of the correlation coefficient (minimum, mean or maximum) to determine whether the overall result of the analysis is robust to the use of the imputed correlation coefficient.

## Results

### Study Selection

**Figure 1:**
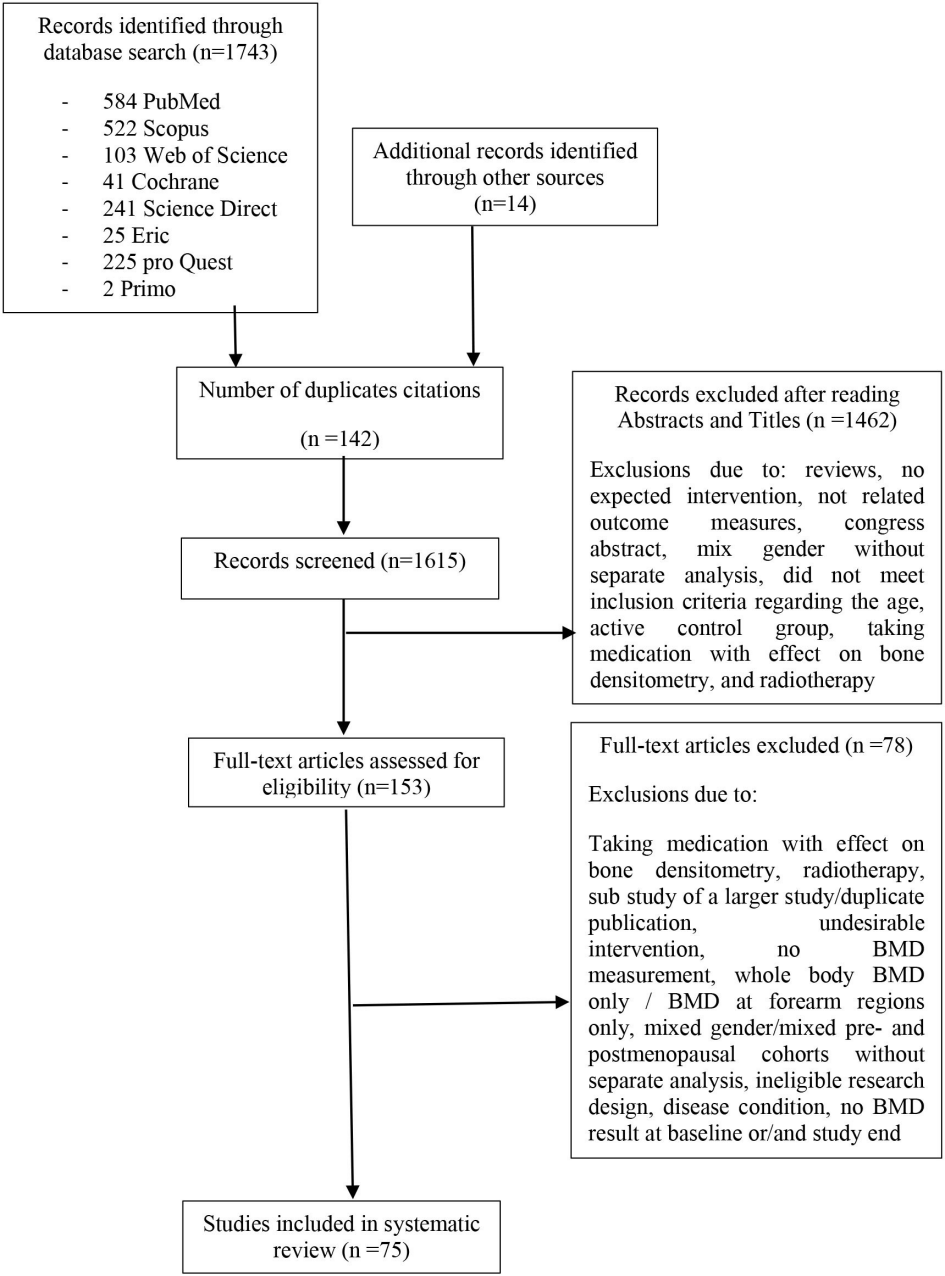
Flow diagram of search process.

Of 1,757 articles initially retrieved, 1,743 studies were found from all included databases and other resources. Duplicate articles were removed and the title and abstract of the remaining articles were screened and checked based on the eligibility criteria. The full-text of 153 potentially relevant articles were then checked, and 78 of them were found not to meet the inclusion criteria. A total of 75 articles were thus included in this study, published from 1989 to 2019 (Figure 1). Three included studies contained English abstracts but with Italian (Tolomio et al., 2009), Portuguese (Orsatti et al., 2013), and German (Kemmler, 1999) full texts, which were translated by native speakers.

### Study and Participants' Characteristic

Seventy-five studies were included in this systematic review and meta-analysis, comprising 88 individual training groups based on our eligibility criteria (Sinaki et al., 1989; Nelson et al., 1991, 1994; Grove and Londeree, 1992; Lau et al., 1992; Pruitt et al., 1992, 1995; Bloomfield et al., 1993; Caplan et al., 1993; Hatori et al., 1993; Martin and Notelovitz, 1993; Bassey and Ramsdale, 1995; Kohrt et al., 1995, 1997; Nichols et al., 1995; Prince et al., 1995; Hartard et al., 1996; Kerr et al., 1996, 2001; Lord et al., 1996; Brooke-Wavell et al., 1997, 2001; Ebrahim et al., 1997; Bassey et al., 1998; Ryan et al., 1998; Adami et al., 1999; Kemmler, 1999; Bemben et al., 2000, 2010; Rhodes et al., 2000; Iwamoto et al., 2001; Chilibeck et al., 2002, 2013; Hans et al., 2002; Sugiyama et al., 2002; Going et al., 2003; Jessup et al., 2003; Milliken et al., 2003; Chan et al., 2004; Kemmler et al., 2004, 2010, 2013; Verschueren et al., 2004; Yamazaki et al., 2004; Englund et al., 2005; Korpelainen et al., 2006; Wu et al., 2006; Evans et al., 2007; Maddalozzo et al., 2007; Woo et al., 2007; Bergstrom et al., 2008; Kwon et al., 2008; Park et al., 2008; Bocalini et al., 2009; Chuin et al., 2009; de Matos et al., 2009; Deng, 2009; Silverman et al., 2009; Tolomio et al., 2009; Sakai et al., 2010; Choquette et al., 2011; Marques et al., 2011b,c; Tartibian et al., 2011; Bolton et al., 2012; Karakiriou et al., 2012; Basat et al., 2013; Orsatti et al., 2013; Bello et al., 2014; Moreira et al., 2014; Liu et al., 2015; Nicholson et al., 2015; Wang et al., 2015; Duff et al., 2016; de Oliveira et al., 2019). The pooled number of participants was 5,300 (intervention group: *n* = 2,901, control group: *n* = 2,399) and sample size in individual studies ranged from five (Grove and Londeree, 1992) to 125 (Adami et al., 1999) participants per group. Table 1 presents a summary of included study characteristics. The mean menopausal age ranged from at least 0.5 (according to eligibility criteria) (Sinaki et al., 1989; Wang et al., 2015) to 24 years (Jessup et al., 2003), and the range of mean ages was between 50 (Bemben et al., 2000) and 79 (Lau et al., 1992; Tella and Gallagher, 2014) years. The mean body mass index (BMI, kg/m^2^) of individual studies varied from 19.7 (Iwamoto et al., 2001) to 32.6 kg/m^2^ (Silverman et al., 2009) (Table 1).

**Table 1:** Participants characteristics of included studies (*n* = 75).

**References**	**Sample size (*n*)**	**Age (years)**	**Menopausal age (years)**	**Body mass (kg)**	**Height (cm)**	**BMI (kg/m^**2**^)**
Adami et al. (1999)	E: 125 C: 125	E: 65 ± 6 C: 63 ± 7	E: 16 ± 7 C: 14 ± 8	n.g. n.g.	n.g. n.g.	E: 24.6 ± 3.3 C: 23.8 ± 3.8
Basat et al. (2013)	RE: 14 HI: 14 C: 14	RE: 56 ± 5 HI: 56 ± 3 C: 56 ± 4	RE: 6 ± 4 HI: 7 ± 2 C: 6 ± 3	n.g. n.g. n.g.	n.g. n.g. n.g.	RE: 25 ± 4.7 HI: 26.4 ± 3.5 C: 27.5 ± 3.7
Bassey et al. (1998)	E: 45 C: 32	E: 56 ± 3 C: 55 ± 4	E: 7 ± 4 C: 5 ± 4	E: 64.7 ± 7.3 C: 66.5 ± 7.8	E: 161 ± 6 C: 163 ± 6	E: 25 ± 2.6 C: 25.1 ± 2.6
Bassey and Ramsdale (1995)	E: 31^a^ C: 32	E: 54 ± 4 C: 55 ± 3	E: 7 ± 4 C: 7 ± 5	E: 63.3 ± 11.4 C: 64.7 ± 6.7	E: 163 ± 6 C: 159 ± 5	E: 24.6 ± 2.7 C: 24.9 ± 3.8
Bello et al. (2014)	E: 10 C: 10	E: 61 ± 6 C: 61 ± 6	n.g. n.g.	n.g. n.g.	n.g. n.g.	n.g. n.g.
Bemben et al. (2010)	E: 22^b^ C: 12	E: 64 ± 1 C: 63 ± 1	>5	E: 76.6 ± 3.2 C: 77.9 ± 4.5	E: 161 ± 2 C: 163 ± 1	E: 30 ± 1 C: 29 ± 1
Bemben et al. (2000)	HR: 11 HL: 13 C: 11	HL: 50 ± 2 HR: 52 ± 2 C: 52 ± 1	HL: 4 ± 1 HR: 2 ± 1 C: 3 ± 1	HL: 74.7 ± 5.6 HR: 62.7 ± 3.4 C: 66.5 ± 4.2	HL: 162 ± 2 HR: 165 ± 2 C: 166 ± 2	HL: 28.7 ± 2.4 HR: 23.2 ± 1.2 C: 24.2 ± 1.7
Bergstrom et al. (2008)	E: 60 C: 52	E: 59 ± 4 C: 60 ± 3	n.g. n.g.	n.g. n.g.	n.g. n.g.	E: 24.4 ± 2.6 C: 24.9 ± 2.3
Bloomfield et al. (1993)	E: 7 C: 7	E: 62 ± 1 C: 59 ± 4	E: 11 ± 3 C: 15 ± 2	E: 77.4 ± 3.5 C: 64.4 ± 2.6	E: 167 ± 2 C: 161 ± 2	E: 28 ± 1.2 C: 25 ± 1
Bocalini et al. (2009)	E: 23 C: 12	E: 69 ± 9 C: 67 ± 8	n.g. n.g.	E: 68 ± 6 C: 69 ± 7	n.g. n.g.	E: 28 ± 4 C: 27 ± 6
Bolton et al. (2012)	E: 19 C: 20	E: 60 ± 6 C: 56 ± 5	E: 13 ± 7 C: 12 ± 7	E: 64.5 ± 9.7 C: 63.6 ± 11.9	E: 160 ± 4 C: 160 ± 6	E: 25.2 ± 4.3 C: 25 ± 4.4
Brooke-Wavell et al. (2001)	E: 18 C: 21	E: 65 ± 3 C: 65 ± 3	>5	E: 68.5 ± 8.9 C: 71.4 ± 12.1	E: 163 ± 7 C: 164 ± 7	n.g. n.g.
Brooke-Wavell et al. (1997)	E: 43 C: 41	E: 65 ± 3 C: 64 ± 3	E: 15 ± 5 C: 15 ± 7	E: 67.7 ± 10.9 C: 67.9 ± 10.6	E: 162 ± 6 C: 163 ± 7	E: 25.8 ± 3.8 C: 25.6 ± 3.5
Caplan et al. (1993)*	E: 19 C: 11	E: 66 ± 1 C: 65 ± 1	E: 18 ± 2 C: 21 ± 3	E: 63.2 ± 2.5 C: 60.6 ± 2.9	E: 158 ± 2 C: 160 ± 2	E: 25.4 ± 0.9 C: 23.5 ± 0.8
Chan et al. (2004)	E: 67 C: 65	E: 54 ± 3 C: 54 ± 3	E: 5 ± 2 C: 4 ± 2	E: 55.4 ± 7.9 C: 54 ± 10.3	E: 150 ± 10 C: 150 ± 20	E: 24.1 ± 4.7 C: 23.5 ± 4.6
Chilibeck et al. (2013)	E+Pl: 86 Pl: 88	E+Pl: 55 ± 6 Pl: 56 ± 7	>1	E+Pl: 73.4 ± 14.1 Pl: 73.6 ± 15.9	E+Pl: 163 ± 5 Pl: 163 ± 6	n.g. n.g.
Chilibeck et al. (2002)*	E: 14 C: 14	E: 57 ± 2 C: 59 ± 2	E: 9 ± 2 C: 8 ± 2	E: 72 ± 4.3 C: 73.2 ± 4.8	E: 164 ± 2 C: 165 ± 1	E: 27 ± 1.7 C: 26.6 ± 1.2
Choquette et al. (2011)	E+Pl: 25 Pl: 26	E+Pl: 58 ± 6 Pl: 59 ± 6	E+Pl: 8 ± 8 Pl: 10 ± 8	E+Pl: 75.4 ± 12.1 Pl: 79.5 ± 9.2	E+Pl: 161 ± 6 Pl: 160 ± 6	E+Pl: 29.1 ± 3.9 Pl: 31 ± 2.9
Chuin et al. (2009)	E+Pl: 11 Pl: 7	E+Pl: 65 ± 3 Pl: 67 ± 4	n.g. n.g.	E+Pl: 66.6 ± 8.5 Pl: 64.2 ± 7.6	n.g. n.g.	E+Pl: 26.5 ± 2.7 Pl: 26 ± 2.8
de Matos et al. (2009)	E: 30 C: 29	E: 57 ± 5 C: 57 ± 5	10 7	E: 59.8 ± 7.6 C: 65 ± 8.3	E: 158 ± 4 C: 159 ± 8	E: 23.9 ± 3.3 C: 25.6 ± 3.1
Deng (2009)	E: 45 C: 36	E: 54 ± 4 C: 51 ± 5	E: 4 ± 3 C: 3 ± 2	E: 58.8 ± 8 C: 58.3 ± 7.5	E: 157 ± 5 C: 159 ± 5	n.g. n.g.
de Oliveira et al. (2019)	E: 17 C: 17	E: 56 ± 7 C: 54 ± 5	E: 8 ± 7 C: 9 ± 7	E: 67.4 ± 8.6 C: 64.6 ± 6.6	E: 157 ± 6 C: 154 ± 4	E: 27.2 ± 2.7 C: 27.3 ± 2.5
Duff et al. (2016)	E: 22 C: 22	E: 65 ± 5 C: 65 ± 5	n.g. n.g.	n.g. n.g.	E: 162 ± 6 C: 160 ± 7	n.g. n.g.
Ebrahim et al. (1997)	E: 81 C: 84	E: 66 ± 8 C: 68 ± 8	n.g. n.g.	n.g. n.g.	n.g. n.g.	E: 26.6 ± 4.3 C: 26.3 ± 4.8
Englund et al. (2005)	E: 24 C: 24	E: 73 ± 4 C: 73 ± 5	n.g. n.g.	E: 66.9 ± 8.7 C: 67.7 ± 8.5	E: 162 ± 6 C: 160 ± 6	E: 25.2 ± 2.7 C: 26.1 ± 3.2
Evans et al. (2007)	E+SP: 11^c^ SP: 10	E+SP: 62 ± 5 SP: 63 ± 5	E+SP: 8 ± 6 SP: 8 ± 5	E+SP: 66.7 ± 13.3 SP: 67.6 ± 7.3	E+SP: 163 ± 7 SP: 161 ± 6	n.g. n.g.
Going et al. (2003)	E: 91 C: 70	E: 56 ± 5 C: 57 ± 5	>3	E: 68.9 ± 11.4 C: 67.8 ± 11.4	E: 163 ± 7 C: 163 ± 5	E: 25.8 ± 3.4 C: 25.5 ± 4
Grove and Londeree (1992)	LI: 5 HI: 5 C: 5	LI: 57 ± 4 HI: 54 ± 2 C: 56 ± 4	LI: 3 ± 2 HI: 4 ± 3 C: 4	LI: 69 ± 12.7 HI: 72.3 ± 19.2 C: 70.5 ± 10.1	n.g. n.g. n.g.	n.g. n.g. n.g.
Hans et al. (2002)	E: 110 C: 35	E: 68 ± 5 C: 66 ± 5	>5	E: 63 ± 7.3 C: 59.5 ± 7.5	E: 161 ± 8 C: 159 ± 8	n.g. n.g.
Hartard et al. (1996)	E: 18 C: 16	E: 64 ± 6 C: 67 ± 10	>2	E: 67 ± 7.7 C: 63.8 ± 11.2	E: 162 ± 7 C: 158 ± 6	n.g. n.g.
Hatori et al. (1993)	E: 23^d^ C: 12	H: 56 ± 4 M: 58 ± 5 C: 58 ± 8	H: 7 ± 5 M: 6 ± 4 C: 9 ± 8	H: 54 ± 5 M: 53.4 ± 6.8 C: 53.9 ± 6	H: 151 ± 3 M: 151 ± 5 C: 151 ± 5	H: 23.3 ± 2.3 M: 23.5 ± 2.4 C: 24.6 ± 3.3
Iwamoto et al. (2001)	E: 8 C: 20	E: 65 ± 5 C: 65 ± 6	E: 16 ± 6 C: 15 ± 6	E: 45.5 ± 6.5 C: 45.8 ± 4	E: 152 ± 8 C: 152 ± 6	E: 19.7 ± 1.3 C: 19.9 ± 2.1
Jessup et al. (2003)	E: 10 C: 10	E: 69 ± 3 C: 69 ± 4	E: 24 ± 11 C: 22 ± 11	E: 78 ± 9.2 C: 84.2 ± 17.7	n.g. n.g.	n.g. n.g.
Karakiriou et al. (2012)*	E: 10 C: 9	E: 53 ± 1 C: 53 ± 1	E: 5 ± 1 C: 3 ± 1	E: 71.2 ± 2.8 C: 75.4 ± 2	E:159 ± 1 C:157 ± 2	E: 28.1 ± 1.1 C: 30.4 ± 0.8
Kemmler et al. (2013)	E: 43 C: 42	E: 52 ± 2 C: 52 ± 3	E: 2 ± 1 C: 2 ± 1	E: 69.5 ± 9.6 C: 70.9 ± 16.8	E: 165 ± 5 C: 165 ± 6	n.g. n.g.
Kemmler et al. (2010)	E: 123 C: 123	E: 69 ± 4 C: 69 ± 4	n.g. n.g.	E: 68.1 ± 10.9 C: 69.5 ± 12	E: 162 ± 6 C: 160 ± 6	n.g. n.g.
Kemmler et al. (2004)	E: 86 C: 51	E: 55 ± 3 C: 56 ± 3	>1	E: 67.6 ± 9.7 C: 64.8 ± 13.6	E: 164 ± 6 C: 162 ± 7	E: 25.1 ± 3.3 C: 24.7 ± 3.9
Kemmler (1999)	E-PM: 15 L-PM: 17 C: 18	EPM: 54 ± 5 LPM: 65 ± 6 C: 56 ± 8	EPM ≤ 8 LPM > 8 C >1	n.g. n.g. n.g.	n.g. n.g. n.g.	EPM: 25.5 ± 4.2 LPM: 26.2 ± 3.8 C: 27.4 ± 5.3
Kerr et al. (2001)	RE: 42 Fit: 42 C: 42	RE: 60 ± 5 Fit: 59 ± 5 C: 62 ± 6	RE: 11 ± 6 Fit: 9 ± 5 C: 12 ± 6	RE: 72.2 ± 12 Fit: 69 ± 11.4 C: 69.3 ± 14.6	RE: 163 ± 5 Fit: 165 ± 6 C: 162 ± 7	n.g. n.g. n.g.
Kerr et al. (1996)	En: 28^e^ S: 28	En: 56 ± 5 S: 58 ± 4	En: 6 ± 4 S: 8 ± 3	En: 70.8 ± 10 S: 69.4 ± 11.4	En: 165 ± 6 S: 165 ± 7	n.g. n.g.
Kohrt et al. (1997) *	JRF: 15 GRF: 18 C: 15	JRF: 65 ± 1 GRF: 66 ± 1 C: 68 ± 1	n.g. n.g. n.g.	JRF: 72.6 ± 2.3 GRF: 70.9 ± 4.2 C: 71.6 ± 1.8	JRF: 164 ± 2 GRF: 163 ± 1 C: 163 ± 2	n.g. n.g. n.g.
Kohrt et al. (1995)	E: 8^f^ C: 8	E: 65 ± 3 C: 66 ± 3	>10	E: 63.4 ± 11.9 C: 63.4 ± 8.1	E: 161 ± 5 C: 161 ± 5	n.g. n.g.
Korpelainen et al. (2006)	E: 84 C: 76	E: 73 ± 1 C: 73 ± 1	n.g. n.g.	E: 61.2 ± 7.9 C: 62.2 ± 9.2	E: 154 ± 5 C: 156 ± 5	E: 25.7 ± 3.4 C: 25.5 ± 3.5
Kwon et al. (2008)	E: 20 C: 20	E: 77 ± 2 C: 77 ± 3	n.g. n.g.	E: 56.4 ± 3.8 C: 58.1 ± 5.6	E: 149 ± 6 C: 152 ± 3	E: 25.9 ± 1.9 C: 25.2 ± 2.8
Lau et al. (1992)	E+Pl: 15 Pl: 15	E+Pl: 79 Pl: 75	n.g. n.g.	n.g. n.g.	n.g. n.g.	n.g. n.g.
Liu et al. (2015)	E: 50 C: 48	E: 63 ± 7 C: 62 ± 8	E: 14 ± 6 C: 13 ± 7	n.g. n.g.	E: 154 ± 4 C: 157 ± 4	n.g. n.g.
Lord et al. (1996)	E: 90 C: 89	E: 72 ± 5 C: 71 ± 5	n.g. n.g.	E: 66 ± 11.4 C: 64.7 ± 14.4	E: 157 ± 6 C:157 ± 7	n.g. n.g.
Maddalozzo et al. (2007)	E: 35 C: 34	E: 52 ± 3 C: 52 ± 3	E: 2 ± 1 C: 2 ± 1	E: 70 ± 8.7 C: 67.1 ± 12.6	n.g. n.g.	n.g. n.g.
Marques et al. (2011b)	E: 30 C: 30	E: 70 ± 5 C: 68 ± 5	n.g. n.g.	n.g. n.g.	n.g. n.g.	E: 28.4 ± 3.7 C: 28.2 ± 3.7
Marques et al. (2011c)	RE: 23 AE: 24 C: 24	RE: 67 ± 5 AE: 70 ± 5 C: 68 ± 6	n.g. n.g. n.g.	n.g. n.g. n.g.	n.g. n.g. n.g.	RE: 28.8 ± 4.6 AE: 27.5 ± 3.8 C: 28.1 ± 3.5
Martin and Notelovitz (1993)	45^min^E: 25 30^min^E: 27 C: 24	45^min^E: 58 ± 7 30^min^E: 60 ± 8 C: 57 ± 7	45^min^ E: 9 ± 9 30^min^E: 13 ± 9 C: 8 ± 7	45^min^ E: 65.6 ± 11.9 30^min^ E: 68.9 ± 11.5 C: 72.9 ± 15.5	45^min^ E: 159 ± 5 30^min^E: 162 ± 7 C: 162 ± 4	n.g. n.g. n.g.
Milliken et al. (2003)	E: 26 C: 30	E: 57 ± 5 C: 57 ± 5	E: 6 ± 3 C: 6 ± 3	E: 68.4 ± 10.6 C: 68.4 ± 10.6	E: 162 ± 6 C: 162 ± 6	n.g. n.g.
Moreira et al. (2014)	E: 64 C: 44	E: 59 ± 7 C: 59 ± 6	>5	E: 73 ± 15.8 C: 74 ± 12.6	E: 157 ± 6 C: 156 ± 6	n.g. n.g.
Nelson et al. (1994)	E: 21 C: 19	E: 61 ± 4 C: 57 ± 6	E: 12 ± 5 C: 10 ± 5	E: 64.7 ± 7.7 C: 62.2 ± 8.9	E: 163 ± 6 C: 164 ± 8	E: 24.4 ± 2.5 C: 23.1 ± 2.2
Nelson et al. (1991)*	E: 21^g^ C: 20	E: 60 ± 1 C: 60 ± 1	E: 11 ± 1 C: 11 ± 1	E: 64 ± 1.4 C: 64 ± 1.4	E: 162 ± 1 C: 162 ± 1	E: 24.4 ± 0.5 E: 24.4 ± 0.5
Nichols et al. (1995)*	E: 17 C: 17	E: 68 ± 2 C: 65 ± 1	E: 18 ± 1 C: 18 ± 1	E: 68.8 ± 2.8 C: 72 ± 13.5	E: 163 ± 1 C: 164 ± 1	n.g. n.g.
Nicholson et al. (2015)	E: 28 C: 29	E: 66 ± 4 C: 66 ± 5	>5	E: 70.6 ± 9.1 C: 66.8 ± 10.7	E: 164 ± 4 C: 163 ± 5	E: 26 ± 3.2 C: 24.5 ± 2.9
Orsatti et al. (2013)	E+Pl: 20 Pl: 20	E+Pl: 56 ± 9 Pl: 55 ± 8	E+Pl: 9 ± 6 Pl: 8 ± 6	n.g. n.g.	n.g. n.g.	E+Pl: 26 ± 3 Pl: 30.4 ± 5.3
Park et al. (2008)	E: 25 C: 25	E: 68 ± 4 C: 68 ± 3	E: 18 ± 2 C: 19 ± 3	n.g. n.g.	E: 153 ± 4 C: 152 ± 4	n.g. n.g.
Prince et al. (1995)	E+Ca: 42 Ca: 42	E+Ca: 63 ± 5 Ca: 62 ± 5	E+Ca: 16 ± 5 Ca: 16 ± 6	n.g. n.g.	n.g. n.g.	n.g. n.g.
Pruitt et al. (1995)	H-int: 15 L-int: 13 C: 12	H-int: 67 L-int: 68 ± 1 C: 70 ± 4	n.g. n.g. n.g.	H-int: 64.5 ± 9.2 L-int: 61.5 ± 4.6 C: 63.8 ± 9.1	H-int: 162 ± 7 L-int: 160 ± 5 C: 160 ± 9	H-int: 24.5 ± 3.4 L-int: 23.9 ± 1.6 C: 25.1 ± 3.1
Pruitt et al. (1992)*	E: 17 C: 10	E: 54 ± 1 C: 56 ± 1	E: 3 C: 4 ± 1	E: 64.2 ± 1.9 C: 65.5 ± 2.9	E: 162 ± 1 C: 163 ± 2	n.g. n.g.
Rhodes et al. (2000)	E: 22 C: 22	E: 69 ± 3 C: 68 ± 3	n.g. n.g.	E: 68.4 ± 12 C: 61.7 ± 12.9	E: 161 ± 5 C: 159 ± 4	n.g. n.g.
Ryan et al. (1998)	E: 18 C: 18	E: 62 ± 6 C: 63 ± 6	>2	E: 79.3 ± 8 C: 83.1 ± 11.3	n.g. n.g.	E: 30.5 ± 2.8 C: 30.9 ± 3
Sakai et al. (2010)*	E: 49 C: 45	E: 68 ± 1 C: 68	n.g. n.g.	E: 51.4 ± 1.1 C: 51.7 ± 0.9	E: 151 ± 1 C: 151 ± 1	E: 22.4 ± 0.4 C: 22.6 ± 0.4
Silverman et al. (2009)	E: 46 C: 40	E: 60 ± 5 C: 58 ± 5	E: 12 ± 8 C: 11 ± 7	E: 84.6 ± 11.3 C: 87.4 ± 14.4	n.g. n.g.	E: 32.1 ± 4.2 C: 32.6 ± 4.6
Sinaki et al. (1989)	E: 34 C: 34	E: 56 ± 4 C: 56 ± 4	>0.5	E: 66.2 ± 9.3 C: 66.1 ± 10.6	E: 163 ± 6 C: 161 ± 5	n.g. n.g.
Sugiyama et al. (2002)*	E: 13^h^ C: 13	E: 52 ± 1 C: 53 ± 1	E: 3 C: 2	E: 54.7 ± 3.4 C: 50.9 ± 1.7	E: 155 ± 2 C: 153 ± 1	E: 22.7 ± 1.2 C: 21.7 ± 0.7
Tartibian et al. (2011)	E: 20 C: 18	E: 61 ± 7 C: 59 ± 8	>8	E: 77.5 ± 10.4 C: 75.9 ± 17.2	E: 167 ± 8 C: 168 ± 16	E: 25.1 ± 7.1 C: 28.5 ± 3.7
Tolomio et al. (2009)	E: 81 C: 79	E: 62 ± 5 C: 64 ± 5	n.g. n.g.	E: 66 ± 10.9 C: 63 ± 9.7	E: 161 ± 10 C: 159 ± 10	n.g. n.g.
Verschueren et al. (2004)	E: 22 C: 24	E: 64 ± 4 C: 64 ± 3	E: 15 ± 6 C: 15 ± 7	E: 70.5 ± 9.6 C: 68.6 ± 14.5	E: 161 ± 6 C: 160 ± 6	E: 27.4 ± 3.5 C: 26.5 ± 5.8
Wang et al. (2015)	TC: 40 TC+RT: 40 C: 39	TC: 58 ± 3 TCRT: 58 ± 3 C: 58 ± 3	>0.5	TC: 60.5 ± 8.3 TCRT: 60 ± 6 C: 60.5 ± 8.3	TC: 159 ± 5 TCRT: 161 ± 4 C: 159 ± 5	n.g. n.g. n.g.
Woo et al. (2007)	TC: 30 RE: 30 C: 30	TC: 70 ± 3 RE: 70 ± 3 C: 69 ± 3	n.g. n.g. n.g.	n.g. n.g. n.g.	n.g. n.g. n.g.	TC: 24.4 ± 4.3 RE: 24.6 ± 4 C: 24.9 ± 3
Wu et al. (2006)	E+Pl: 34 Pl: 34	E+Pl: 55 ± 3 Pl: 55 ± 3	E+Pl: 4 ± 2 Pl: 4 ± 2	E+Pl: 54.1 ± 7.3 Pl: 51.4 ± 7.1	E+Pl: 155 ± 6 Pl: 157 ± 6	E+Pl: 22.4 ± 2.9 Pl: 20.9 ± 2.2
Yamazaki et al. (2004)*	E: 32 C: 18	E: 64 ± 3 C: 66 ± 3	E: 17 ± 2 C: 15 ± 2	E: 51.2 ± 1.4 C: 50.1 ± 1.6	E: 155 ± 1 C: 156 ± 1	E: 21.2 ± 0.7 C: 21.1 ± 1.1

a According to the text, 63 women were randomized equally.

b It is not stated, seven drop out belong to which groups.

c It is not stated, nine drop out belong to which groups.

d It is not clear to which exercise groups two persons who failed to complete the program belong.

e One side of body is considered as control and the other side as intervention.

f No data concerning participants/group; we assumed an equal allocation.

g Exercise with or without 831 mg/d Ca vs. sedentary control with or without 831 mg/d Ca.

h According to the baseline table in the article, there are 13 PMW in the exercise group, however, the text said that six persons in exercise groups were excluded due to low compliance with exercise but it is not clear whether these participants are in the pre- or post-menopausal group.

AE, aerobic exercise; C, control; Ca,calcium; E, exercise; En, Endurance; EPM, early post-menopausal; Fit, fitness; GRF, ground-reaction forces (i.e., walking); H, High; HI, high impact; H-int, high intensity; HL, high load; HR, high repetition; JRF, joint-reaction forces; LI, low impact; L-int, Low intensity; LPM, late post-menopausal; M, Moderate; n.g., not given; Pl, Placebo; RE, resistance exercise; S, Strength; SP, soy protein; TCRT, Tai Chi resistance training; TC, Tai Chi; All values are presented as mean ± SD, otherwise it is stated; *Numbers are presented as mean ± SE. Eligibility criteria with respect to post-menopausal age were utilized, if the studies provided no information regarding this item.

Twenty-seven studies recruited participants with sedentary life style (Nelson et al., 1991, 1994; Grove and Londeree, 1992; Bloomfield et al., 1993; Kohrt et al., 1995, 1997; Brooke-Wavell et al., 1997, 2001; Ryan et al., 1998; Adami et al., 1999; Rhodes et al., 2000; Iwamoto et al., 2001; Jessup et al., 2003; Yamazaki et al., 2004; Wu et al., 2006; Woo et al., 2007; Bocalini et al., 2009; Kemmler et al., 2010; Choquette et al., 2011; Marques et al., 2011b,c; Tartibian et al., 2011; Karakiriou et al., 2012; Orsatti et al., 2013; Bello et al., 2014; Moreira et al., 2014; de Oliveira et al., 2019), 33 trials involved participants with some kinds of exercises activities (Pruitt et al., 1992, 1995; Martin and Notelovitz, 1993; Bassey and Ramsdale, 1995; Nichols et al., 1995; Prince et al., 1995; Hartard et al., 1996; Kerr et al., 1996, 2001; Lord et al., 1996; Ebrahim et al., 1997; Bassey et al., 1998; Kemmler, 1999; Bemben et al., 2000, 2010; Chilibeck et al., 2002, 2013; Going et al., 2003; Milliken et al., 2003; Chan et al., 2004; Kemmler et al., 2004, 2013; Bergstrom et al., 2008; Kwon et al., 2008; Park et al., 2008; Deng, 2009; Silverman et al., 2009; Sakai et al., 2010; Bolton et al., 2012; Basat et al., 2013; Nicholson et al., 2015; Wang et al., 2015; Duff et al., 2016), while the remaining studies did not provide any information with respect to the life style status of participants (Sinaki et al., 1989; Lau et al., 1992; Caplan et al., 1993; Hatori et al., 1993; Hans et al., 2002; Sugiyama et al., 2002; Verschueren et al., 2004; Englund et al., 2005; Korpelainen et al., 2006; Evans et al., 2007; Maddalozzo et al., 2007; Chuin et al., 2009; de Matos et al., 2009; Tolomio et al., 2009; Liu et al., 2015).

Sixty-one studies comprised healthy participants (Sinaki et al., 1989; Nelson et al., 1991; Grove and Londeree, 1992; Lau et al., 1992; Pruitt et al., 1992, 1995; Bloomfield et al., 1993; Caplan et al., 1993; Hatori et al., 1993; Martin and Notelovitz, 1993; Bassey and Ramsdale, 1995; Kohrt et al., 1995, 1997; Nichols et al., 1995; Prince et al., 1995; Kerr et al., 1996, 2001; Lord et al., 1996; Brooke-Wavell et al., 1997, 2001; Ebrahim et al., 1997; Bassey et al., 1998; Ryan et al., 1998; Adami et al., 1999; Kemmler, 1999; Bemben et al., 2000, 2010; Rhodes et al., 2000; Chilibeck et al., 2002, 2013; Sugiyama et al., 2002; Going et al., 2003; Jessup et al., 2003; Milliken et al., 2003; Chan et al., 2004; Verschueren et al., 2004; Englund et al., 2005; Wu et al., 2006; Evans et al., 2007; Maddalozzo et al., 2007; Woo et al., 2007; Kwon et al., 2008; Park et al., 2008; Bocalini et al., 2009; Chuin et al., 2009; Deng, 2009; Silverman et al., 2009; Kemmler et al., 2010, 2013; Sakai et al., 2010; Choquette et al., 2011; Marques et al., 2011b,c; Tartibian et al., 2011; Orsatti et al., 2013; Bello et al., 2014; Moreira et al., 2014; Nicholson et al., 2015; Wang et al., 2015; Duff et al., 2016; de Oliveira et al., 2019), and the remaining studies recruited participants with osteopenia, osteoporosis, or with a history of spinal fracture(s) (Nelson et al., 1994; Hartard et al., 1996; Iwamoto et al., 2001; Hans et al., 2002; Kemmler et al., 2004; Yamazaki et al., 2004; Korpelainen et al., 2006; Bergstrom et al., 2008; de Matos et al., 2009; Tolomio et al., 2009; Bolton et al., 2012; Karakiriou et al., 2012; Basat et al., 2013; Liu et al., 2015) (Table 2).

**Table 2:** Exercise prescription characteristics of included studies (*n* = 75).

**References**	**Status**	**Length months**	**PR- INT**	**Main part of exercise**	**SiSp**	**Volume (min/w), Supervision(Attendance)**	**Exercise/strain composition**	**Summary of main part of exercise**
Adami et al. (1999)	Healthy 16 ± 7 y post Sedentary	6	No	DRT (focus on forearm sites); volleyball in a sitting/standing position	No Yes	2 × 95–110, SJE (83%) 7 × 30 HE (n.g.)	SJE: 15–30 min warm up (walking), 70 min press-up, volleyball, 10 min DRT for the forearm with a 500 g weight. Number of reps (10–25)/min increased progressively. HE: Repeat all exercise	L-Intensity AET and RT (forearm site)
Basat et al. (2013)	Osteopenia 6 ± 4 y post No-BSE	6	No	DRT (focus on lower body with few trunk exercises)	Yes Yes	3 × 60, S-JE (>60%)	15 min warm up (walking, cycling), 30–40 min RT: ≥9 exercises, one set, 10 reps (more details n.g.)	L/M-intensity DRT
		6	No	Rope skipping	No Yes	7 × 35, S-JE (>60%)	15 min warm up (walking, cycling), Maximum 50 jumps/session (more details n.g.)	M-Impact jumping
Bassey et al. (1998)	Healthy 7 ± 4 y post No vigorous Ex > 1 h/w	12	No	Jumping: counter-movement jumps (CMJ)	No Yes	5 × 10, HE 1 × 10, S-JE (91%)	50 CMJ barefoot with both legs, five sets × 10 reps with ground reaction forces (GRF): 4 × body mass	H-Impact jumping
Bassey and Ramsdale (1995)	Healthy 7 ± 4 y post No-BSE	12	No	Heel-drops, jumping, skipping	No Yes	1 × ?, S-JE 7 × ?, HE (84%)	HE: 50 heel-drops barefoot on a thinly covered floor with knee and hip extended. S-JE: jumping and skipping (More details n.g.)	H-Impact heel drop
Bello et al. (2014)	Healthy 61 ± 6 y No-M/H intensity Ex >20 min or 2/w	8	No	Walking; DRT (all main muscle groups); aquatic exercise (RT main muscle groups)	Yes Yes	3 × 40-?, S-JE (85%)	40 min walking 1 × w, WB-circuit training 1 × w with easy loads: six exercises, three sets, 15–20 reps. Aquatic exercise 1 × w: four exercise, three sets, 15–20 reps; all at RPE 12–15 of Borg CR 20. 1 × w each type of exercise	L-Intensity WB AET and L-Intensity DRT
Bemben et al. (2010)	Healthy >5 y post No-RT	8	No	DRT (all main muscle groups) with machines	Yes Yes	3 × ≈60, S-JE (90%)	5 min warm up (walking, cycling), eight exercises, three sets, 10 reps, 80% 1RM + dumbbell wrist curls and seated abdominal flexion L/M intensity	H-Intensity DRT
Bemben et al. (2000)	Healthy 3 ± 1 y post No-RT	6	Yes	DRT (all main muscle groups) with machines	Yes Yes	3 × 60, S-JE (87%)	DRT:45 min, 8 exercises, three sets, eight reps, 80% 1RM	H-Intensity DRT
		6	Yes	DRT (all main muscle groups) with machines	Yes Yes	3 × 60, S-JE (93%)	DRT: 45 min, eight exercises, three sets, 16 reps, 40% 1RM	L-Intensity DRT
Bergstrom et al. (2008)	Osteopenia (forearm fractures) 59 ± 4 y No-BSE	12	Yes	DRT (all main muscle groups); AET; walking	Yes Yes	1–2 × 60, S-JE 3 × 30, HE HT and S-JE (95%)	S-JE: 25 min DRT, 25 min WB-AET (more details n.g.) HE: fast walking (more details n.g.)	L-Intensity AET and ?-Intensity DRT
Bloomfield et al. (1993)	Healthy 11 ± 3 y post Sedentary	8	Yes	Cycle ergometer	No No	3 × 50, S-JE (82%)	15 min warm up [flexibility and calisthenics (more details n.g.)], 30 min cycling at 60–80% HRmax, 5 min walking (cool down)	H-Intensity Non-WB AET
Bocalini et al. (2009)	Healthy >8 y post Sedentary	6	Yes	DRT (all main muscle groups)	Yes Yes	3 × 60, S-JE (>90%)	10 min warm up (low impact running), 12 exercises, three sets, 10 reps, 85% 1RM with focus on eccentric exercises, 1 min rest (alternate upper and lower body exercises) between ex	H-Intensity DRT
Bolton et al. (2012)	Osteopenia 13 ± 7 y post No-BSE	12	Yes	DRT (muscle groups n.g.: “loading the proximal femur”); jumping	No Yes	3 × 60, S-JE 1/w (88%) Daily HT	S-JE: 40 min (?) exercises, two sets, eight reps, 80% 1RM with slow velocity, one set with reduced load and high velocity (12 rep). HT: Daily three sets, 10 reps of jumps (more details n.g.)	M/H-Impact and H-Intensity DRT
Brooke-Wavell et al. (2001)	Healthy >5 y post Sedentary	12	No	Brisk walking	No Yes	>3 × >20 (140 min/w), non-supervised (>90%)	4–5 × 25–35 min/d ≈ 70% HRmax	M-Intensity WB-AET
Brooke-Wavell et al. (1997)	Healthy 15 ± 6 y post Sedentary	12	No	Brisk walking	No Yes	140 min/w, Non-supervised (100%)	20–50 min long for each walk, ≈ 70% HRmax	M-Intensity WB-AET
Caplan et al. (1993)	Healthy 18 ± 8 y post n.g.	24	No	Aerobic dance, ball games; DRT: floor exercises (more details n.g.)	? Yes	2 × 60, S-JE (n.g.)≥1 ×20–30, HT (n.g.)	20–25 min AET, 10 min ball games (more details n.g.) 20–30 min DRT (more details n.g.)	L-Impact, ?-Intensity WB-AET and ?-Intensity DRT
Chan et al. (2004)	Healthy 5 ± 2 y post No >0.5 h/w	12	No	Tai Chi: Yang Style [all main muscle groups (more details n.g.)]	? Yes	5 × 50, S-JE (≈84%)	Slow, smooth movements with constant velocity	Tai Chi (Yang Style)
Chilibeck et al. (2013)	Healthy >1 y post No-BSE	24	Yes	Walking; DRT (all main muscle groups) on machines	Yes Yes	2 × n.g., S-JE 4 × 20–30, HT and S-JE (77%)	S-JE: 15 exercises, two sets, eight reps, 80% 1RM HT and S-JE: walking at 70% HRmax	M-Intensity WB-AET and H-Intensity DRT
Chilibeck et al. (2002)	Healthy 9 ± 2 y post No-vigorous Ex	12	Yes	DRT (all main muscle groups) on machines	Yes Yes	3 × ?, S-JE (78%)	12 exercises, two sets, 8–10 reps, ≈70% 1RM	H-Intensity DRT
Choquette et al. (2011)	Healthy 8 ± 8 y post Sedentary	6	Yes	Treadmill and cycling; DRT (all main muscle groups) on machines and with free weights	Yes Yes	3 × 60, S-JE (≥85%)	AET: 30 min at 40–85% HRmax; after 3 months H-intensity intervals of 4 × 4 min ≥90% HRmax, 3 min rest at 50–65% HRmax. RT: 30 min, ?exercise, one set, 12–15 rep increased to four sets 4–6 reps, at 60–85%1RM	H-Intensity AET and H-Intensity DRT
Chuin et al. (2009)	Healthy >8 y post n.g.	6	Yes	DRT (most main muscle groups) on machines	Yes Yes	3 × 60, S-JE (>90%)	15 min warm up (treadmill/cycle ergometer), DRT: 45 min, eight exercises, three sets, eight reps at 80% 1RM, rest between sets 90–120 s, 1RM-test each 4 weeks	H-Intensity DRT
de Matos et al. (2009)	≥Osteopenia 10 y post n.g.	12	Yes	DRT (all main muscle groups) on machines or free weights; AET (Bike, Treadmill)	Yes Yes	3 × 45–65, n.g. (presumably S-JE) (n.g.)	WB-/non-WB-AET (Bike, treadmill, Stepper): 5–20 min (RPE 4–6 on Borg CR 10). DRT: 30–40 min, nine exercises,? sets, 10–15 reps, ? 1RM, TUT: three s conc-3 s eccentric; 1 min rest between sets and exercise	L/M-Intensity DRT and M-Intensity AET
Deng (2009)	Healthy 4 ± 3 y post No-BSE	12	Yes	Brisk walking, stepping, jumping; DRT (all main muscle groups) on machines with free weights	Yes Yes	2 × 60, S-JE 3–5 × 60, HE (82%)	S-EJ: 45 min DRT, nine exercises, 2–5 sets, 12–40 reps, at 50–60% 1RM, self-selected rest (more details n.g.). HE: 30 min walking, at 50–80% HRmax, 15 min step routine, 50–300 jumps from a 4 inch bench	H-Impact, H-Intensity WB-AET, M-Intensity DRT
de Oliveira et al. (2019)	Healthy 8 ± 7 y post Sedentary	6	Yes	Pilates (all main muscle groups) on machines	Yes Yes	3 × 60, S-JE (93%)	21 exercises (strengthening and flexibility), one set, 10 reps, 1 min rest between exercises, 5–6 at Borg CR10	M-Intensity DRT
Duff et al. (2016)	Healthy >8 y post No-RT	9	Yes	DRT (all main muscle groups) on machines and with free weights	Yes Yes	3 × ?, S-JE (84%)	12 exercises, two sets, 8–12 reps to muscular fatigue, ? 1RM (more details n.g.)	?-Intensity DRT
Ebrahim et al. (1997)	Healthy (upper limb fractures) 66 ± 8 y No limit	24	No	Brisk walking	No Yes	3 × 40, HE (100%)	40 min walking, “faster than usual, but not so fast as to be uncomfortable”	L-Intensity WB-AET
Englund et al. (2005)	Healthy >8 y post n.g.	12	Yes	Walking/jogging; DRT (all main muscle groups)	Yes Yes	2 × 50, S-JE (67%)	WB-AET: 10 min warm up, 15 min walking/jogging. DRT: 12 min, two sets, 8–12 reps., ? 1RM (more details n.g.)	L/M-Intensity WB-AET and ?-Intensity DRT
Evans et al. (2007)	Healthy ≈8 ± 6 y post n.g.	9	Yes	Walking/running, rowing, stair-climbing (machines)	Yes Yes	3 × 45, S-JE (n.g.)	WB and Non-WB AET (machines) at 55–80% VO_2_peak. Rest by changing exercise mode	H-Intensity WB-AET
Going et al. (2003)	Healthy 3–11 y post No-RT, <120 min Ex	12	Yes	Walking, Jogging, skipping, hopping, stepping with weighted vests; DRT (all main muscle groups) on machines with free weights	Yes Yes	3 × ≈60, S-JE (72%)	10 min warm up (walking), 20–25 min WB-AET at 60% HRmax, 120–300 stair/steps with 5–13 kg weighted vest. DRT: 7 exercises, two sets, 6–8 reps 70–80% 1 RM	L-Intensity WB-AET and H-Intensity DRT
Grove and Londeree (1992)	Healthy 4 ± 3 y post Sedentary	12	No	Jumping variations, heel drops (GRF≥2x body mass)	No Yes	3 × 60, S-JE (83%)	20 min of high impact exercises. 15 min cool down (RT with abdominal and leg adduction/abduction exercises)	H-Impact intensity WB-AET
		12	No	Walking, charleston, heel jacks (GRF <1.5 × body mass)	No Yes	3 × 60, S-JE (80%)	20 min of low impact exercises. 15 min cool down (RT with abdominal and leg adduction/abduction exercises)	L-Impact intensity WB-AET
Hans et al. (2002)	≥Osteopenia >5 y post n.g.	24	Yes (?)	Heel-drops: barefoot on a force measuring platform (osteocare)	No Yes	5 × 3–5, HE (65%)	Impact loading: strength or height 25–50% above the estimated resting force, daily 120 correct force impacts	L-Impact intensity WB-AET
Hartard et al. (1996)	Osteopenia >2 y post <1 h/w, No-BSE	6	Yes	DRT (all main muscle groups) on machines	Yes Yes	2 × ?, S-JE (>83%)	14 exercises, 1–2 sets, 8–12 reps, 70% 1RM, TUT: concentric: 3–4 s–eccentric 3–4s. ≥2 min rest between sets	M-Intensity DRT
Hatori et al. (1993)	Healthy ≈7 ± 5 y post n.g.	7	No	Walking below the anaerobic threshold at “flat grass covered ground”	No Yes	3 × 30, n.g.(n.g.)	30 min walking at 90% anaerobic threshold HR (6.2 km/h)	L/M-Intensity WB-AET
		7	No	Walking above the anaerobic threshold at “flat grass covered ground”	No Yes	3 × 30, n.g.(n.g.)	30 min walking at 110% anaerobic threshold HR (7.2 km/h)	H-Intensity WB-AET
Iwamoto et al. (2001)	Osteoporosis 16 ± 6 y post Sedentary	24	Yes	Walking; DRT (“Gymnastics”: lower limbs and trunk exercises)	Yes Yes	Daily (walking) × ?, HE 2 × daily RTx?,HE (n.g.)	Additionally (to basic activity walking) ≈3,000 steps/d, RT: ≥ 4 exercises, two sets, 15 reps, ?% 1RM	L-Intensity WB-AET and ?-Intensity DRT
Jessup et al. (2003)	Healthy >8 y post Sedentary	8	Yes	Walking, stairclimbing; DRT (most main muscle groups) on machines	Yes Yes	3 × 60–90, S-JE (n.g.)	DRT: 20–35 min, eight exercises, ? sets, 8–10 reps, 50–75% 1RM. WB-AET: 30–45 min with weighted vest (increased up to 10% body-mass)	?-Intensity WB-AE and M-Intensity DRT
Karakiriou et al. (2012)	Osteopenia 5 ± 2 y post Sedentary	6	No	Step aerobic exercise; DRT (all main muscle groups)	Yes Yes	2 × ? RT, S-JE 1 × 45 min AET (80%)	15 min warm up (walking on treadmill/cycling ergometer and jumping). Abdominal and back extension exercises (one exercise for each muscle group, 2–4 sets of 16 repetitions). RT:11 exercises, 2–3 sets, 10–12 reps at 70% 1RM, 30 s rest between exercises, 3 min between sets. AET: 20 min, nine exercise, two circuits of 40 s; rest: 20 s between exercises, 2 min between circuits, 70–85% HRmax	M/H-Impact WB-AET and H-Intensity DRT
Kemmler et al. (2013)	Healthy 2 ± 1 y post No-BSE	12	Yes	Block periodized AET, jumping; isometric and DRT (all main muscle groups) exercise on machines with free weight, body mass	Yes Yes	3 × 45–60, S-JE (67%)	Block I: 1 × 45 min/w H-Impact aerobic 75–85% HRmax, 2 × 20 min/w aerobic 75–85% HRmax, 4 × 15–20 jumps, 90 s rest. RT: 15 min, 8–12 floor exercises (trunk, hip, legs), 1–2 sets, rep?, 30 s rest. RT: 20 min, eight exercises, two sets, 8–9 rep, 45 s rest up, TUT: 2s concentric, 2 s eccentric. to 80% 1RM	H-Impact; H-Intensity WB-AET and H-Intensity DRT
Kemmler et al. (2010)	Healthy >8 y post Sedentary	18	Yes	Aerobic dance; DRT (all main muscle groups)	Yes Yes	2 × 60, S-JE (76%) 2 × 20, HE (42%)	AET: 20 min at 70–85% HRmax. RT: 10–15 exercises, 1–3 sets of 6–10 s maximum isometric contractions, 20–30 s rest, 3 upper body exercises, 2–3 sets 10–15 reps, TUT: 2s concentric, 2s eccentric at 65–70% 1RM; three lower extremity exercises, two sets eight reps, 1 min rest at 80% 1RM. HT: RT 1–2 sets, 6–8 exercise, 10–15 rep. 2–3 belt exercises, two sets, 10–15 rep	H-Intensity WB-AET and H-Intensity DRT
Kemmler et al. (2004)	Osteopenia 1–8 y post No-BSE	26	Yes	Fast walking and running, jumping; DRT (all main muscle groups) on machines with free weight, body mass	Yes Yes	2 × 60–70, S-JE (79%) 2 × 25, HT (61%)	AET: 20 min at 65–85% HRmax. Jumping started after 5–6 months with 4x 15 multi-lateral jumps. DRT: 30–40 min, 1/w. The first 6 month: 13 ex, two sets, 20–12 rep, TUT: 2 s concentric, 2 s eccentric at 50–65% RM, 90 s rest between sets and exercises. Then, 12 w blocks of H-intensity at 70–90% 1RM interleaved by 4 w at 55–79% 1RM. Isometric RT: 30–40 min, 1/w, 12–15 exercises (trunk and femur), 2–4 sets, 15–20 rep, 15–20 s rest. HT: rope skipping (three set, 20 rep), RT	H-Impact, H-Intensity WB-AET, and H-Intensity DRT
Kemmler (1999)	Healthy 1–15 y post No-BSE	9	Yes	Running, gaming, jumping; DRT (all main muscle groups)	Yes Yes	2 × 90, S-JE (82%) 2 × 35, HT (59%)	AET: 25 min at 70–80% HRmax. RT: 65 min, 12–15 exercises, 2–4 sets of 8 s maximum isometric contractions; six trunk, upper back, lower extremity exercises, 20–25 reps at 60–65% 1 RM. HT: resistance exercises	H-Impact, H-Intensity WB-AET and M-Intensity DRT
Kerr et al. (2001)	Healthy ≈10 ± 6 y post <2 h/w	24	Yes	DRT (all main muscle groups)	Yes Yes	3 × 60, S-JE (74%)	≈30 min brisk walking and stretching, RT: 30 min, nine exercises, three sets at 8 RM (≈75–80% 1RM)	H-Intensity DRT
		24	No	DRT (all main muscle groups); Stationary cycling	Yes Yes	3 × 60, S-JE (77%)	≈30 min brisk walking and stretching. RT: 30 min, nine exercises, three set, eight rep, 40 s/exercise with “minimal load”; 10 s rest between the exercises (more details n.g.). Stationary cycling 40 s, HR <150 beats/min	L-Intensity DRT and Non-WB-AET
Kerr et al. (1996)	Healthy ≈7 ± 4 y post No-RT, no racquet sports, No-Ex > 3 h/w	12	Yes	Unilateral DRT (all main muscle groups, randomized allocation of the left side or right side to exercise or control group) on machines or free weights	Yes Yes	3 × 45–60, S-JE (89%)	13 exercises, three sets at 20 RM, 3–5 rep (≈60–65% 1RM), 2–3 min rest between sets	M-Intensity DRT
		12	Yes	Unilateral DRT (see above)	Yes Yes	3 × 20–30, S-JE (87%)	13 exercises, three sets at 8 RM, 3–5 rep (≈75–80% 1RM), 2–3 min rest between sets	H-Intensity DRT
Kohrt et al. (1997)	Healthy >8 y post Sedentary	11	Yes	Walking, jogging, stair climbing	No Yes	3–5 × 30–45, n.g. (pre-sumably S-JE) (≈70%)	First 2 months flexibility, 9 months WB at 60–85% HRmax	H-Intensity WB-AET
		11	Yes	DRT (all main muscle groups) with free weights and on machines; rowing	Yes Yes	3–5 × 40–60, n.g. (presumably S-JE) (≈70%)	First 2 months flexibility, DRT: 2/w, ≈20–30 min, eight exercises, 2–3 sets, 8–12 reps “to fatigue” (≈70–80% 1RM). Rowing: 3/w,15–30 min, 2–3 sets × 10 min at 60–85% HRmax	H-Intensity DRT and Non WB-AET
Kohrt et al. (1995)	Healthy >8 y post Sedentary	11	Yes	Walking, jogging, stair climbing	No Yes	3–5 × 45, HE (≈70%)	First 2 months flexibility, 9 months WB: 5–10 min warm up (treadmill 60–70% HRmax), 30 min WB at 65–85% HRmax	H-Intensity WB-AET
Korpelainen et al. (2006)	Osteopenia >8 y post n.g.	30	Yes	Jumping, walking/jogging, dancing, stamping, chair climbing	Yes Yes	1 × 60, S-JE 7 × 20, HE (≈75%)	S-JE: 45 min WB-AET. The first six months: 1 × 60 min S-JE and daily × 20 min HE. The second 6 months: HE: daily × 20 min HE applying the same exercise to S-JE	M/H-Impact and H-Intensity WB-AET
Kwon et al. (2008)	Healthy >8 y post No-Ex>2/w	6	Yes RT?	Aerobic dance; DRT (six upper and lower body exercises) with free weights	Yes Yes	3 × 80, n.g. (presumably S-JE) (n.g.)	30 min AET at 40–75% HRmax, 30 min DRT of 6 exercises, ? sets, 3–10 reps to voluntary fatigue (i.e., 75% 1RM)	M-Intensity WB-AET and M/H-Intensity DRT
Lau et al. (1992)	Healthy >8 y post n.g.	10	No	Stepping up and down, Upper trunk movements	Yes Yes	4 × ≈20–25, S-JE (n.g.)	100 steps on a 23 cm block 15 min upper trunk movements (?) in a standing position with sub-maximum effort (more details n.g.)	M-Intensity WB-AET
Liu et al. (2015)	Osteoporosis 14 ± 6 y post n.g.	12	No	Tai-Chi	No Yes	3 × daily ≈3–5, HE (96%)	Eight exercise brocade, seven rep (raising slowly the arms coming on the toes stretching the back and go back on the heel with arms hanging down)	Tai-Chi
Lord et al. (1996)	Healthy >8 y post No equal intensity with the intervention	12	No	Conditioning period: Brisk walking, multilateral stepping, lunges, heel rises; DRT (all main muscle groups) using owns body mass	Yes Yes	2 × 60, S-JE (73%)	5 min warm up (paced walking), conditioning period 35–40 min: AET and guided functional gymnastics for all main muscle groups (sets?, reps?, intensity?)	L/M-Intensity WB-AET and ?-Intensity DRT
Maddalozzo et al. (2007)	Healthy 1–3 y post n.g.	12	Yes	DRT (back squat, deadlifts) with free weights	Yes Yes	2 × 50, S-JE (85%)	15–20 min warm up (exercise focusing on posture, muscle engagement, abdominal strength, flexibility) two sets, 10–12 reps, 50% 1RM. Main part: 20–25 min, two exercises, three sets, 8–12 reps, 60 s rest between sets at 60–75% 1RM, TUT: 1–2 s concentric, 2–3 s eccentric	M-Intensity DRT
Marques et al. (2011b)	Healthy >8 y post Sedentary	8	Yes	Marching, bench stepping, heel-drops; DRT (most main muscle groups) with weighted vests, elastic bands, free weights	Yes Yes	2 × 60, S-JE (72%)	15 min WB-AET with Peak-GRF up to 2.7 × body mass and high strain frequency (120–125 beats/min), 10 min for ≥7 muscle endurance exercises, 1–3 sets, 8–15 reps, ?1RM (more details n.g.), 10 min balance and dynamic exercise (walking, playing with ball, rope, sticks, etc.), 10 min agility training (coordination, balance, ball games, dance)	M/H-Intensity WB-AET and L/M-Intensity DRT
Marques et al. (2011c)	Healthy >8 y post Sedentary	8	Yes	Walking, stepping, skipping, jogging, dancing	Yes Yes	3 × 60, S-JE (78%)	Only the first 6 w 10 min DRT (lower body). 35–40 min of WB-AET (50–85% HRR) with Peak-GRF up to 2.7 × body mass with up to 120 beats/min	H-Intensity WB-AET
		8	Yes	DRT (all main muscle groups) on machines	Yes Yes	3 × 60, S-JE (78%)	8–10 min warm up (cycling/rowing ergometer) at low intensity. 30–40 min DRT, 8 exercises, two sets, 15–6 reps, 50–80% 1RM with variable TUT (3–6s/rep.), 120 s rest between sets, 5–10 min cool down (walking and stretching)	H-Intensity DRT
Martin and Notelovitz (1993)	Healthy ≈11 ± 9 y post No-BSA	12	Yes	Brisk walking on treadmill	No Yes	3 × 36–40, n.g. (presumably S-JE) (79%)	30 min brisk walking (4–6.2 km/h at 3–7% incline) at 70–85% HRmax	H-Intensity WB-AET
		12	Yes	Brisk walking on treadmill	No Yes	3 × 51–55, n.g. (presumably S-JE) (82%)	45 min brisk walking (4–6.2 km/h at 3–7% incline) at 70–85% HRmax	H-Intensity WB-AET
Milliken et al. (2003)	Healthy 6 ± 3 y post <2 h/w	12	Yes	Walking, skipping, multilateral stepping, jumping with weighted vests; DRT (all main muscle groups) with free weights, on machines; functional gymnastics	Yes Yes	3 × 75, S-JE (n.g.)	20 min WB-AET at 50–70% HRmax. 35 min DRT: 8 exercises, two sets, 6–8 reps, 70–80% 1 RM. Functional gymnastics for shoulder and abdominals using elastic bands and physio-balls	M-Impact, M-Intensity WB-AET, H-Intensity DRT
Moreira et al. (2014)	Healthy >5 y post Sedentary	6	Yes	Aquatic exercise (RT and AET in 1.1–1.3 m water depth) without equipment	Yes Yes	3 × 50–60, S-JE (85%)	2–5 sets of 30–10 s of four upper and lower body exercise with maximum effort and movement speed (full ROM), 1–1:40 min rest, 16–9 min at 55–90% HRmax	H-Intensity aquatic RT and AET
Nelson et al. (1994)	Healthy (6 women with 1 spine fracture) 12 ± 5 y post Sedentary	12	Yes	DRT (most main muscle groups) on machines	Yes Yes	2 × 55, S-JE (88%)	45 min, five exercises, three sets, eight reps, 50- 80% 1RM, TUT-6–9 s/rep, 3 s rest between reps, 90–120 s rest between sets	H-Intensity DRT
Nelson et al. (1991)	Healthy 11 ± 1 y post Sedentary	12	No	Walking with weighted vest	No Yes	4 × 50, S-JE (90%)	Walking with a 3.1 kg weighted vest at 75–80% HRmax	H-Intensity WB-AET
Nichols et al. (1995)	Healthy >8 y post ≥3 × 30min/w	12	Yes	DRT (all main muscle groups) on machines	Yes Yes	3 × ≈45–60, S-JE (82%)	5 min warm up (walking), 8 exercises, 1–3 sets, 10–12 reps, 50–80% 1RM; 30–60s rest between exercises, 60 s rest between sets	H-Intensity DRT
Nicholson et al. (2015)	Healthy >5 y post No-RT	6	Yes	DRT (all main muscle groups): “Body Pump Release 83” (i.e., barbell exercises)	Yes Yes	2 × 50, S-JE(89%)	10 × up to 6 min blocks of exercises for all main muscle groups (21 exercises in total); up to 108 reps (squats), ≤ 30% 1RM	very L-Intensity DRT
Orsatti et al. (2013)	Healthy 9 ± 6 y post Sedentary	9	Yes	DRT (all main muscle groups) with free weights and on machines	Yes Yes	3 × 50–60, S-JE (n.g.)	Eight exercises three sets, 8–15 reps at 40–80% 1RM, three sets-−20–30 reps for trunk flexion and calf raises, 1–2 min rest between sets	H-intensity DRT
Park et al. (2008)	Healthy >8 y post ≤ 7 h/w M-Ex	12	No	WB-AET; RT (more details n.g.)	? Yes	3 × 60, n.g. (n.g.)	10 min RT, 23 min of WB exercise at 65–70% HRmax (more details n.g.)	M-Intensity WB-AET and ?-Intensity RT
Prince et al. (1995)	Healthy >8 y post ≤ 2 h/w Ex	24	No	WB-AET (more details n.g.)	No Yes	4 × 60, 2 × S-JE/2 × HE (39%)	4 × WB exercise (including 2 × walking) at 60% HRmax (more details n.g.)	L-Intensity WB-AET
Pruitt et al. (1995)	Healthy >8 y post No-RT	12	Yes	DRT (all main muscle groups) on machines	Yes Yes	3 × 55–65, S-JE (81%)	50–55 min, 10 exercises, one warm up set, 14 reps, at 40% 1 RM, two sets, seven reps, 80% 1RM	H-Intensity DRT
	Healthy >8 y post No-RT	12	Yes	DRT (all main muscle groups) on machines	Yes Yes	3 × 55–65, S-JE (77%)	50–55 min, 10 exercises, three sets, 14 reps, at 40% 1RM	L-Intensity DRT
Pruitt et al. (1992)	Healthy 3 ± 1 y post No-BSE	9	Yes	DRT (all main muscle groups) with free weights and on machines	Yes Yes	3 × 60, S-JE (83%)	40 min, 11 exercises, one set, at 10–12 RM for upper body and 10–15 RM for lower body (more details n.g.)	H-Intensity DRT
Rhodes et al. (2000)	Healthy >8 y post Sedentary	12	Yes	DRT (all main muscle groups) on machines	Yes Yes	3 × 60, S-JE (85%)	10 min warm up (cycle ergometer), DRT: 40 min, ≥6 exercises, three set, eight reps, 75% 1RM, TUT: 2–3 s concentric−3–4 s eccentric movement/rep applied in a circuit mode	H-Intensity DRT
Ryan et al. (1998)	Healthy >2 y post Sedentary	6	Yes	Walking, jogging on treadmill	No Yes	3 × 55, S-E (>90%)	Up to (4th month) 35 min walking/jogging at 50–70% VO_2_max, 10 min cool down (cycle ergometer), Energy-intake restriction of 250–350 kcal/d (weight loss study).	H-Intensity WB-AET
Sakai et al. (2010)	Healthy >8 y post n.g.	6	No	Unilateral standing on one leg	No Yes	7 × 2, HE (≥70%)	Three sets (early, at noon, in the evening) of unilateral standing for 1 min on each leg with eyes open	WB-AET and Balance
Silverman et al. (2009)	Healthy 12 ± 8 y post Sedentary	6	No	Walking	No Yes	3 × 45–60, S-JE >1 session(78%)	walking at 50–75% HRmax, energy-intake restriction of 250–350 kcal/d (weight loss study)	M-Intensity WB-AET
Sinaki et al. (1989)	Healthy >0.5 y post n.g.	24	Yes	DRT (back strengthening exercise in a prone position using a back pack; ≈hyperextensions) with free weights	Yes No	5 × ?, HE (n.g.)	One back strengthening exercise, one set, 10 reps, with a weight equivalent to 30% of the maximum isometric back muscle strength in pounds (maximum 23 kg)	L/M-Intensity DRT
Sugiyama et al. (2002)	Healthy 3 y post n.g.	6	No	Rope skipping (more details n.g.)	No Yes	2–3 × ?, HE (82%)	100 jump/session (more details n.g.)	M/H-Impact jumping
Tartibian et al. (2011)	Healthy >8 y post Sedentary	6	Yes	Walking/jogging on treadmill	No Yes	3–6 × 25–45, S-JE (95%)	First 12 weeks: 3–4 × 25–30 min at 45–55% HRmax, second 12 weeks: 4–6 × 40–45 min at 55–65% HRmax	L/M-Intensity WB-AET
Tolomio et al. (2009)	≥Osteopenia 2–22 y post n.g.	11	No	DRT (joint mobility, elastic bands, balls); aquatic exercise (more details n.g.)	? Yes	3 × 60, S-JE and 1 × HE (n.g.)	The first 11 w only in gym, then two times in gym and once in water. 15 min warm up (brisk walking, stretching), 2 × 30 min/week RT, 1 × 30 min/week water gymnastics (more details n.g.). two periods (6 and 10 w) training at home (more details n.g.)	?-Intensity DRT and aquatic exercise
Verschueren et al. (2004)	Healthy 15 ± 6 y post n.g.	6	Yes	DRT (leg press, leg extension)	No Yes	3 × 60, n.g. (presumably S-JE) (n.g.)	20 min warm up (running, stepping, or cycling) at 60–80% HRmax, DRT:2 exercise, 1–3 set, 20–8 rep	H-Intensity DRT
Wang et al. (2015)	Healthy >0.5 y post No Tai Chi	12	No	Tai Chi (Yang-style)	? Yes	2 × 60, S-JE 2 × 60, Group E with video (n.g.)	40 min: 5 reps × 6 min set, 42 type compositions each, 2 min rest (more details n.g.)	Tai Chi (Yang-Style)
		12	No	Tai Chi-RT (includes 4 Chen style actions)	? Yes	2 × 60, S-JE 2 × 60, Group E with video (n.g.)	40 min: 6 reps × 5min exercise, 2 min rest (more details n.g.)	Tai-Chi-RT (includes 4 Chen style actions)
Woo et al. (2007)	Healthy >8 y post Sedentary	12	No	Tai-Chi (Yang Style)	? Yes	3 × ?, S-JE (81%)	24 forms of Yang-Style Tai Chi	Tai Chi (Yang-style)
		12	No	DRT (arm-lifting, hip abduction, heel raise, hip-flexion,-extension, squat) using elastic bands	Yes Yes	3 × ?, S-JE (76%)	Six exercises, 30 reps (no more information given)	L/M-Intensity DRT
Wu et al. (2006)	Healthy 4 ± 2 y post Sedentary	12	No	Walking	No Yes	3 × 60, S-JE (n.g.)^*^	45 min of walking with 5–6 km/h	L-Intensity WB-AET
Yamazaki et al. (2004)	≥Osteopenia 17 ± 8 y post Sedentary	12	No	Walking	No Yes	≥4 × 60, n.g. (presumably HE) (100%)	8,000 steps/session at 50% VO_2_max	M-Intensity WB-AET

* *Obviously low, according to the additional number steps/day compared with the sedentary control group. AET, aerobic exercise training; BSE, Bone specific exercise; DRT, dynamic resistance training; GRF, Ground Reaction Forces; HE, Home Exercise; JE, joint exercise program; PS, Partially supervised; PR-INT, Progression of intensity parameters; PrInt, Progression of Intensity; RPE, rate of perceived exertion; S, Supervised; SiSp, Site specifity (for LS and hip ROI); ?, no clear information; WB, weight bearing; TUT, time under tension; L, low; M, moderate; H, high. Status: We focus on osteoporosis/osteopenia and fractures reported only. Otherwise subjects were considered “healthy”; Period of menopausal status: In the case of no information, the mean age was reported; Physical activity: Predominately we used the characterization of the authors. In some cases (e.g., Martin and Notelovitz, 1993) we summarize the information given to no bone specific exercise (no BSE); Progression: We only consider the progression of exercise intensity; Type of exercise: We subsume the information given in weight-bearing (WB) vs. Non-WB aerobic exercise training (AET); resistance (RT) or dynamic resistance exercise (DRT), jumping, aquatic exercise or Tai Chi; Site specifity (SiSp): First line: Estimated site specific of the exercise type on LS-BMD; Second line: Estimated site specific of the exercise type on FN-BMD. E.g., we considered the effect of walking as site specific for FN but not for LS. Depending on the exercises applied, DRT was considered as site specific for both BMD-ROIs; Exercise volume/week; setting, attendance: Number of sessions per week × minutes per session (e.g., 3 × 60); setting of the exercise application, i.e., either supervised group exercise (S-JE) or home exercise or exercise individually performed without supervision (HE). In parenthesis: Attendance as defined as rate of sessions performed (%); Composition of strain/exercise parameters per session: AET: specific exercise (i.e., walking, jogging, aerobic dance), exercise duration, exercise intensity; DRT: exercises/number of exercises; number of sets, number of repetitions; exercise intensity; jumping: type of jumps, number of jumps, intensity of jumps; Tai-Chi: style, number of forms. ¤We did not include warm up in the table, if the authors did not report the duration and type of exercise as warm-up; cycle ergometer ≤ 5 min as warm-up, stretching and balance as cool-down have not been included in the table*.

### Exercise Characteristic Description

Table 2 outlines the exercise prescription characteristics. The program duration ranged from six (Hartard et al., 1996; Ryan et al., 1998; Adami et al., 1999; Bemben et al., 2000; Sugiyama et al., 2002; Verschueren et al., 2004; Kwon et al., 2008; Bocalini et al., 2009; Chuin et al., 2009; Silverman et al., 2009; Sakai et al., 2010; Choquette et al., 2011; Tartibian et al., 2011; Karakiriou et al., 2012; Basat et al., 2013; Moreira et al., 2014; Nicholson et al., 2015; de Oliveira et al., 2019) to 30 months (Korpelainen et al., 2006).

Eleven studies applied an intervention period of ≥18 months (Sinaki et al., 1989; Caplan et al., 1993; Prince et al., 1995; Ebrahim et al., 1997; Iwamoto et al., 2001; Kerr et al., 2001; Hans et al., 2002; Kemmler et al., 2004, 2010; Korpelainen et al., 2006; Chilibeck et al., 2013), 39 trials used an intervention period between 9 and 18 months (Nelson et al., 1991, 1994; Grove and Londeree, 1992; Lau et al., 1992; Pruitt et al., 1992, 1995; Martin and Notelovitz, 1993; Bassey and Ramsdale, 1995; Kohrt et al., 1995, 1997; Nichols et al., 1995; Kerr et al., 1996; Lord et al., 1996; Brooke-Wavell et al., 1997, 2001; Bassey et al., 1998; Kemmler, 1999; Rhodes et al., 2000; Chilibeck et al., 2002; Going et al., 2003; Milliken et al., 2003; Chan et al., 2004; Yamazaki et al., 2004; Englund et al., 2005; Wu et al., 2006; Evans et al., 2007; Maddalozzo et al., 2007; Woo et al., 2007; Bergstrom et al., 2008; Park et al., 2008; de Matos et al., 2009; Deng, 2009; Tolomio et al., 2009; Bolton et al., 2012; Kemmler et al., 2013; Orsatti et al., 2013; Liu et al., 2015; Wang et al., 2015; Duff et al., 2016), and 25 scheduled a short intervention period of ≤ 8 months (Bloomfield et al., 1993; Hatori et al., 1993; Hartard et al., 1996; Ryan et al., 1998; Adami et al., 1999; Bemben et al., 2000, 2010; Sugiyama et al., 2002; Jessup et al., 2003; Verschueren et al., 2004; Kwon et al., 2008; Bocalini et al., 2009; Chuin et al., 2009; Silverman et al., 2009; Sakai et al., 2010; Choquette et al., 2011; Marques et al., 2011b,c; Tartibian et al., 2011; Karakiriou et al., 2012; Basat et al., 2013; Bello et al., 2014; Moreira et al., 2014; Nicholson et al., 2015; de Oliveira et al., 2019). Of importance, no study reported a delay between the end of the intervention and the control assessments.

Of all 75 included studies, 13 had two intervention groups (based on our eligibility criteria). Five of them assigned various types of exercises between the intervention groups (Grove and Londeree, 1992; Kohrt et al., 1997; Woo et al., 2007; Marques et al., 2011c; Basat et al., 2013), the other 5 trials compared two different training intensities (Hatori et al., 1993; Pruitt et al., 1995; Kerr et al., 1996, 2001; Bemben et al., 2000) whereas, Martin and Notelovitz (1993) categorized intervention groups according to the training duration (Martin and Notelovitz, 1993). Moreover, one study considered two intervention groups with different Tai Chi styles (Wang et al., 2015). Kemmler (1999) classified participants based on the menopausal status, and they were included in the analysis as individual intervention groups.

The majority of the 88 intervention groups employed aerobic exercise as the main component of their intervention, with walking and/or jogging the most common types (Nelson et al., 1991; Grove and Londeree, 1992; Lau et al., 1992; Bloomfield et al., 1993; Hatori et al., 1993; Martin and Notelovitz, 1993; Bassey and Ramsdale, 1995; Kohrt et al., 1995, 1997; Prince et al., 1995; Brooke-Wavell et al., 1997, 2001; Ebrahim et al., 1997; Bassey et al., 1998; Ryan et al., 1998; Hans et al., 2002; Sugiyama et al., 2002; Yamazaki et al., 2004; Korpelainen et al., 2006; Wu et al., 2006; Evans et al., 2007; Silverman et al., 2009; Sakai et al., 2010; Marques et al., 2011c; Tartibian et al., 2011; Basat et al., 2013). Twenty-six training protocols combined aerobic and resistance exercise (Caplan et al., 1993; Lord et al., 1996; Kohrt et al., 1997; Adami et al., 1999; Kemmler, 1999; Iwamoto et al., 2001; Kerr et al., 2001; Going et al., 2003; Jessup et al., 2003; Milliken et al., 2003; Kemmler et al., 2004, 2010, 2013; Englund et al., 2005; Bergstrom et al., 2008; Kwon et al., 2008; Park et al., 2008; de Matos et al., 2009; Deng, 2009; Choquette et al., 2011; Marques et al., 2011b; Bolton et al., 2012; Karakiriou et al., 2012; Chilibeck et al., 2013; Bello et al., 2014; Moreira et al., 2014). Resistance exercise as the predominant component was prescribed by 27 intervention groups (Sinaki et al., 1989; Pruitt et al., 1992, 1995; Nelson et al., 1994; Nichols et al., 1995; Hartard et al., 1996; Kerr et al., 1996, 2001; Kohrt et al., 1997; Bemben et al., 2000, 2010; Rhodes et al., 2000; Chilibeck et al., 2002; Verschueren et al., 2004; Maddalozzo et al., 2007; Woo et al., 2007; Bocalini et al., 2009; Chuin et al., 2009; Marques et al., 2011c; Basat et al., 2013; Orsatti et al., 2013; Nicholson et al., 2015; Duff et al., 2016; de Oliveira et al., 2019), Tai Chi was utilized in 5 training groups (Chan et al., 2004; Woo et al., 2007; Liu et al., 2015; Wang et al., 2015).

Exercise intensities varied considerably between the exercise protocols (very low to high; Garber et al., 2011). With respect to resistance training, most of the studies prescribed a training intensity of 70–80% of one repetition maximum (1-RM). Aerobic exercise was predominately performed in the range between 60 and 80% of the maximum heart rate maximum (HRmax). In 54 intervention groups, the exercise intensity was progressively increased during the intervention period (Sinaki et al., 1989; Pruitt et al., 1992, 1995; Bloomfield et al., 1993; Martin and Notelovitz, 1993; Nelson et al., 1994; Kohrt et al., 1995, 1997; Nichols et al., 1995; Hartard et al., 1996; Kerr et al., 1996, 2001; Ryan et al., 1998; Kemmler, 1999; Bemben et al., 2000; Rhodes et al., 2000; Iwamoto et al., 2001; Chilibeck et al., 2002, 2013; Hans et al., 2002; Going et al., 2003; Jessup et al., 2003; Milliken et al., 2003; Kemmler et al., 2004, 2010, 2013; Verschueren et al., 2004; Englund et al., 2005; Korpelainen et al., 2006; Evans et al., 2007; Maddalozzo et al., 2007; Bergstrom et al., 2008; Kwon et al., 2008; Bocalini et al., 2009; Chuin et al., 2009; de Matos et al., 2009; Deng, 2009; Choquette et al., 2011; Marques et al., 2011b,c; Tartibian et al., 2011; Bolton et al., 2012; Orsatti et al., 2013; Moreira et al., 2014; Nicholson et al., 2015; Duff et al., 2016; de Oliveira et al., 2019).

Fifty-one intervention groups adequately addressed their endpoints LS and/or FN BMD by their exercise protocol (site specificity) (Lau et al., 1992; Pruitt et al., 1992, 1995; Nelson et al., 1994; Nichols et al., 1995; Hartard et al., 1996; Kerr et al., 1996, 2001; Lord et al., 1996; Kohrt et al., 1997; Kemmler, 1999; Bemben et al., 2000, 2010; Rhodes et al., 2000; Iwamoto et al., 2001; Chilibeck et al., 2002, 2013; Going et al., 2003; Jessup et al., 2003; Milliken et al., 2003; Kemmler et al., 2004, 2010, 2013; Englund et al., 2005; Korpelainen et al., 2006; Evans et al., 2007; Maddalozzo et al., 2007; Woo et al., 2007; Bergstrom et al., 2008; Kwon et al., 2008; Bocalini et al., 2009; Chuin et al., 2009; de Matos et al., 2009; Deng, 2009; Choquette et al., 2011; Marques et al., 2011b,c; Karakiriou et al., 2012; Basat et al., 2013; Orsatti et al., 2013; Bello et al., 2014; Moreira et al., 2014; Nicholson et al., 2015; Duff et al., 2016; de Oliveira et al., 2019). Some studies defined BMD at LS and/or FN as a study endpoint—however, the corresponding bone regions were not (or at least not adequately) addressed by their training protocol (Table 2).

The majority of studies prescribed an exercise frequency of three times per week (range 2–9 sessions/week) (Nelson et al., 1994; Hartard et al., 1996; Lord et al., 1996; Adami et al., 1999; Iwamoto et al., 2001; Englund et al., 2005; Maddalozzo et al., 2007; Marques et al., 2011b; Nicholson et al., 2015). Exercise session duration ranged from ≈2 to 110 min (Adami et al., 1999; Sakai et al., 2010). During resistance training sessions 1–21 exercises (Sinaki et al., 1989; Nicholson et al., 2015; de Oliveira et al., 2019), with up to 108 repetitions (Nicholson et al., 2015) structured in 1–5 sets (Sinaki et al., 1989; Pruitt et al., 1992; Deng, 2009; Basat et al., 2013; de Oliveira et al., 2019), were applied per session. Sixteen RT studies (Nelson et al., 1994; Nichols et al., 1995; Hartard et al., 1996; Kerr et al., 1996; Kemmler et al., 2004, 2010, 2013; Maddalozzo et al., 2007; Bocalini et al., 2009; Chuin et al., 2009; de Matos et al., 2009; Marques et al., 2011c; Karakiriou et al., 2012; Orsatti et al., 2013; Moreira et al., 2014; de Oliveira et al., 2019) additionally listed rest period between sets and/or exercises (range: 15–180 s). Time under tension (TUT) was reported in nine studies only (Nelson et al., 1994; Hartard et al., 1996; Rhodes et al., 2000; Kemmler et al., 2004, 2010, 2013; Maddalozzo et al., 2007; de Matos et al., 2009; Marques et al., 2011c) and ranged between 3 and 9 s per repetition, with two studies using fast or explosive movements in the concentric part of the exercise.

Exercise sessions were supervised in 59 studies (Nelson et al., 1991, 1994; Grove and Londeree, 1992; Lau et al., 1992; Pruitt et al., 1992, 1995; Bloomfield et al., 1993; Caplan et al., 1993; Martin and Notelovitz, 1993; Bassey and Ramsdale, 1995; Nichols et al., 1995; Prince et al., 1995; Hartard et al., 1996; Kerr et al., 1996, 2001; Lord et al., 1996; Bassey et al., 1998; Ryan et al., 1998; Adami et al., 1999; Kemmler, 1999; Bemben et al., 2000, 2010; Rhodes et al., 2000; Chilibeck et al., 2002, 2013; Going et al., 2003; Jessup et al., 2003; Milliken et al., 2003; Chan et al., 2004; Kemmler et al., 2004, 2010, 2013; Englund et al., 2005; Korpelainen et al., 2006; Wu et al., 2006; Evans et al., 2007; Maddalozzo et al., 2007; Woo et al., 2007; Bergstrom et al., 2008; Kwon et al., 2008; Bocalini et al., 2009; Chuin et al., 2009; Deng, 2009; Silverman et al., 2009; Tolomio et al., 2009; Choquette et al., 2011; Marques et al., 2011b,c; Tartibian et al., 2011; Bolton et al., 2012; Karakiriou et al., 2012; Basat et al., 2013; Orsatti et al., 2013; Bello et al., 2014; Moreira et al., 2014; Nicholson et al., 2015; Wang et al., 2015; Duff et al., 2016; de Oliveira et al., 2019). Ten trials used non-supervised home-exercise protocols (Sinaki et al., 1989; Kohrt et al., 1995; Brooke-Wavell et al., 1997, 2001; Ebrahim et al., 1997; Iwamoto et al., 2001; Hans et al., 2002; Sugiyama et al., 2002; Sakai et al., 2010; Liu et al., 2015). The remaining studies did not state the corresponding setting comprehensively (Hatori et al., 1993; Kohrt et al., 1997; Verschueren et al., 2004; Yamazaki et al., 2004; Park et al., 2008; de Matos et al., 2009).

The majority of studies reported attendance rates of more than 70% [minimum: 39% (Prince et al., 1995), maximum: 100% (Brooke-Wavell et al., 1997; Ebrahim et al., 1997; Yamazaki et al., 2004)]. However, 15 studies did not provide any information regarding the attendance rate (Sinaki et al., 1989; Lau et al., 1992; Hatori et al., 1993; Iwamoto et al., 2001; Jessup et al., 2003; Milliken et al., 2003; Verschueren et al., 2004; Wu et al., 2006; Evans et al., 2007; Kwon et al., 2008; Park et al., 2008; de Matos et al., 2009; Tolomio et al., 2009; Orsatti et al., 2013; Wang et al., 2015).

### Methodological Quality

PEDro scores are listed in Table 3. The methodological quality of 14 trials can be considered as high (Ebrahim et al., 1997; Chilibeck et al., 2002, 2013; Jessup et al., 2003; Korpelainen et al., 2006; Woo et al., 2007; Park et al., 2008; Kemmler et al., 2010, 2013; Bolton et al., 2012; Orsatti et al., 2013; Nicholson et al., 2015; Duff et al., 2016; de Oliveira et al., 2019), 44 studies demonstrated moderate (Sinaki et al., 1989; Nelson et al., 1991, 1994; Grove and Londeree, 1992; Lau et al., 1992; Pruitt et al., 1992, 1995; Caplan et al., 1993; Hatori et al., 1993; Martin and Notelovitz, 1993; Nichols et al., 1995; Prince et al., 1995; Hartard et al., 1996; Kerr et al., 1996, 2001; Brooke-Wavell et al., 1997, 2001; Kemmler, 1999; Rhodes et al., 2000; Iwamoto et al., 2001; Hans et al., 2002; Going et al., 2003; Milliken et al., 2003; Chan et al., 2004; Verschueren et al., 2004; Wu et al., 2006; Evans et al., 2007; Maddalozzo et al., 2007; Bergstrom et al., 2008; Bocalini et al., 2009; Chuin et al., 2009; Tolomio et al., 2009; Bemben et al., 2010; Sakai et al., 2010; Choquette et al., 2011; Marques et al., 2011b,c; Tartibian et al., 2011; Basat et al., 2013; Bello et al., 2014; Moreira et al., 2014; Liu et al., 2015; Wang et al., 2015), while the remaining studies (*n* = 17) were classified as being of low quality (Table 3).

**Table 3:** Assessment of risk of bias for included studies (*n* = 75).

**References**	**Eligibility criteria**	**Random allocation**	**Allocation concealment**	**Inter group homogeneity**	**Blinding subjects**	**Blinding personnel**	**Blinding assessors**	**participation≥ 85% allocation**	**Intention to treat analysis^**a**^**	**Between group comparison**	**Measure of variability**	**Total score**
Adami et al. (1999)	Y	0	0	1	0	0	0	1	0	1	1	4
Basat et al. (2013)	Y	1	1	1	0	0	0	0	0	1	1	5
Bassey et al. (1998)	Y	1	0	1	0	0	0	0	0	1	1	4
Bassey and Ramsdale (1995)	Y	1	0	1	0	0	0	0	0	1	1	4
Bello et al. (2014)	Y	1	0	1	0	0	0	0	1	1	1	5
Bemben et al. (2010)	Y	0	0	1	0	0	0	1	1	1	1	5
Bemben et al. (2000)	Y	1	0	1	0	0	0	0	0	1	1	4
Bergstrom et al. (2008)	Y	1	1	1	0	0	0	0	1	1	1	6
Bloomfield et al. (1993)	Y	0	0	0	0	0	0	1	1	1	1	4
Bocalini et al. (2009)	Y	1	0	1	0	0	1	0	0	1	1	5
Bolton et al. (2012)	Y	1	1	0	0	0	1	1	1	1	1	7
Brooke-Wavell et al. (2001)	Y	0	0	1	0	0	0	1	1	1	1	5
Brooke-Wavell et al. (1997)	Y	1	0	1	0	0	0	1	0	1	1	5
Caplan et al. (1993)	Y	0	0	1	0	0	0	1	1	1	1	5
Chan et al. (2004)	Y	1	0	1	0	0	0	0	1	1	1	5
Chilibeck et al. (2013)	Y	1	1	1	0	0	1	1	1	1	1	8
Chilibeck et al. (2002)	Y	1	1	1	1	1	0	0	1	1	1	8
Choquette et al. (2011)	Y	1	0	1	0	0	0	0	1	1	1	5
Chuin et al. (2009)	Y	1	0	1	0	0	0	0	1	1	1	5
de Matos et al. (2009)	Y	0	0	1	0	0	0	0	0	1	1	3
Deng (2009)	Y	0	0	1	0	0	0	1	0	1	1	4
de Oliveira et al. (2019)	Y	1	1	1	0	0	1	1	1	1	1	8
Duff et al. (2016)	Y	1	1	1	1	0	1	0	1	1	1	8
Ebrahim et al. (1997)	Y	1	1	1	0	0	1	0	1	1	1	7
Englund et al. (2005)	Y	1	0	1	0	0	0	0	0	1	1	4
Evans et al. (2007)	Y	1	1	1	0	0	0	0	1	1	1	6
Going et al. (2003)	Y	1	0	1	0	0	0	0	1	1	1	5
Grove and Londeree (1992)	Y	1	0	1	0	0	0	1	1	1	1	6
Hans et al. (2002)	Y	1	0	1	0	0	0	0	1	1	1	5
Hartard et al. (1996)	Y	0	0	1	0	0	0	1	1	1	1	5
Hatori et al. (1993)	Y	1	0	1	0	0	1	1	0	1	1	6
Iwamoto et al. (2001)	Y	1	0	1	0	0	0	0	1	1	1	5
Jessup et al. (2003)	Y	1	1	0	0	0	1	1	1	1	1	7
Karakiriou et al. (2012)	Y	1	0	0	0	0	0	0	0	1	1	3
Kemmler et al. (2013)	Y	1	0	1	1	0	1	0	1	1	1	7
Kemmler et al. (2010)	Y	1	1	1	1	0	1	1	1	1	1	9
Kemmler et al. (2004)	Y	0	0	1	0	0	0	0	1	1	1	4
Kemmler (1999)	Y	0	0	1	0	0	0	1	1	1	1	5
Kerr et al. (2001)	Y	1	0	1	0	0	0	0	1	1	1	5
Kerr et al. (1996)	Y	1	0	1	0	0	0	0	1	1	1	5
Kohrt et al. (1997)	Y	0	0	1	0	0	0	0	1	1	1	4
Kohrt et al. (1995)	Y	0	0	1	0	0	0	0	1	1	1	4
Korpelainen et al. (2006)	Y	1	1	1	0	0	1	0	1	1	1	7
Kwon et al. (2008)	Y	0	0	1	0	0	0	0	0	1	1	3
Lau et al. (1992)	Y	1	1	1	0	0	0	0	1	0	1	5
Liu et al. (2015)	Y	1	0	1	0	0	0	1	1	1	1	6
Lord et al. (1996)	Y	1	0	1	0	0	0	0	1	1	1	5
Maddalozzo et al. (2007)	Y	1	0	1	0	0	0	1	1	1	1	6
Marques et al. (2011b)	Y	1	1	1	0	0	0	0	1	1	1	6
Marques et al. (2011c)	Y	1	1	1	0	0	0	0	1	1	1	6
Martin and Notelovitz (1993)	Y	1	0	1	0	0	0	0	1	1	1	5
Milliken et al. (2003)	Y	1	0	1	0	0	0	1	1	1	1	6
Moreira et al. (2014)	Y	1	0	1	0	0	0	1	1	1	1	6
Nelson et al. (1994)	Y	1	0	1	0	0	0	1	1	1	1	6
Nelson et al. (1991)	Y	0	0	1	0	0	0	1	1	1	1	5
Nichols et al. (1995)	Y	1	0	1	0	0	0	0	1	1	1	5
Nicholson et al. (2015)	Y	1	1	1	0	0	0	1	1	1	1	7
Orsatti et al. (2013)	Y	1	1	1	0	0	0	1	1	1	1	7
Park et al. (2008)	Y	1	1	1	0	0	0	1	1	1	1	7
Prince et al. (1995)	Y	1	1	1	0	0	0	0	1	1	1	6
Pruitt et al. (1995)	Y	1	0	1	0	0	0	0	1	1	1	5
Pruitt et al. (1992)	Y	0	0	1	0	0	0	1	1	1	1	5
Rhodes et al. (2000)	Y	1	0	1	0	0	0	1	1	1	1	6
Ryan et al. (1998)	Y	0	0	1	0	0	0	0	1	1	1	4
Sakai et al. (2010)	Y	1	1	1	0	0	0	1	0	1	1	6
Silverman et al. (2009)	Y	0	0	1	0	0	1	0	0	1	1	4
Sinaki et al. (1989)	Y	1	0	1	0	0	0	1	1	1	1	6
Sugiyama et al. (2002)	Y	0	0	1	0	0	0	0	0	1	1	3
Tartibian et al. (2011)	Y	1	0	1	0	0	0	1	1	1	1	6
Tolomio et al. (2009)	Y	1	0	1	0	0	0	1	0	1	1	5
Verschueren et al. (2004)	Y	1	1	1	0	0	1	0	0	1	1	6
Wang et al. (2015)	Y	1	0	1	0	0	0	1	1	1	1	6
Woo et al. (2007)	Y	1	1	1	0	0	1	1	1	1	1	8
Wu et al. (2006)	Y	1	0	1	1	0	0	0	1	1	1	6
Yamazaki et al. (2004)	Y	0	0	1	0	0	0	0	1	1	1	4

a *The point is awarded not only for intention to treat analysis, but also when “all subjects for whom outcome measures were available received the treatment or control condition as allocated”. Mainly higher scores were hindered by the lack of allocation concealment, subject, therapies and assessor blinding, and reporting the key outcomes for ≥85% of subjects as the common limitations*.

### Outcomes Measures

Fourteen of the 75 trials assessed BMD at LS and proximal femur (Prince et al., 1995; Pruitt et al., 1995; Bemben et al., 2000, 2010; Chilibeck et al., 2002, 2013; Sugiyama et al., 2002; Kemmler et al., 2004; Wu et al., 2006; Maddalozzo et al., 2007; Choquette et al., 2011; Nicholson et al., 2015; Duff et al., 2016; de Oliveira et al., 2019), 9 studies measured BMD only at LS (Sinaki et al., 1989; Grove and Londeree, 1992; Hatori et al., 1993; Martin and Notelovitz, 1993; Iwamoto et al., 2001; Verschueren et al., 2004; Yamazaki et al., 2004; Evans et al., 2007; Karakiriou et al., 2012), while seven studies focused only on the BMD of at least one proximal femur ROI (Kerr et al., 1996; Hans et al., 2002; Korpelainen et al., 2006; Tolomio et al., 2009; Sakai et al., 2010; Marques et al., 2011c; Bello et al., 2014).

### Meta-Analysis Results

#### Effect of Exercise on BMD at the LS

**Figure 2:**
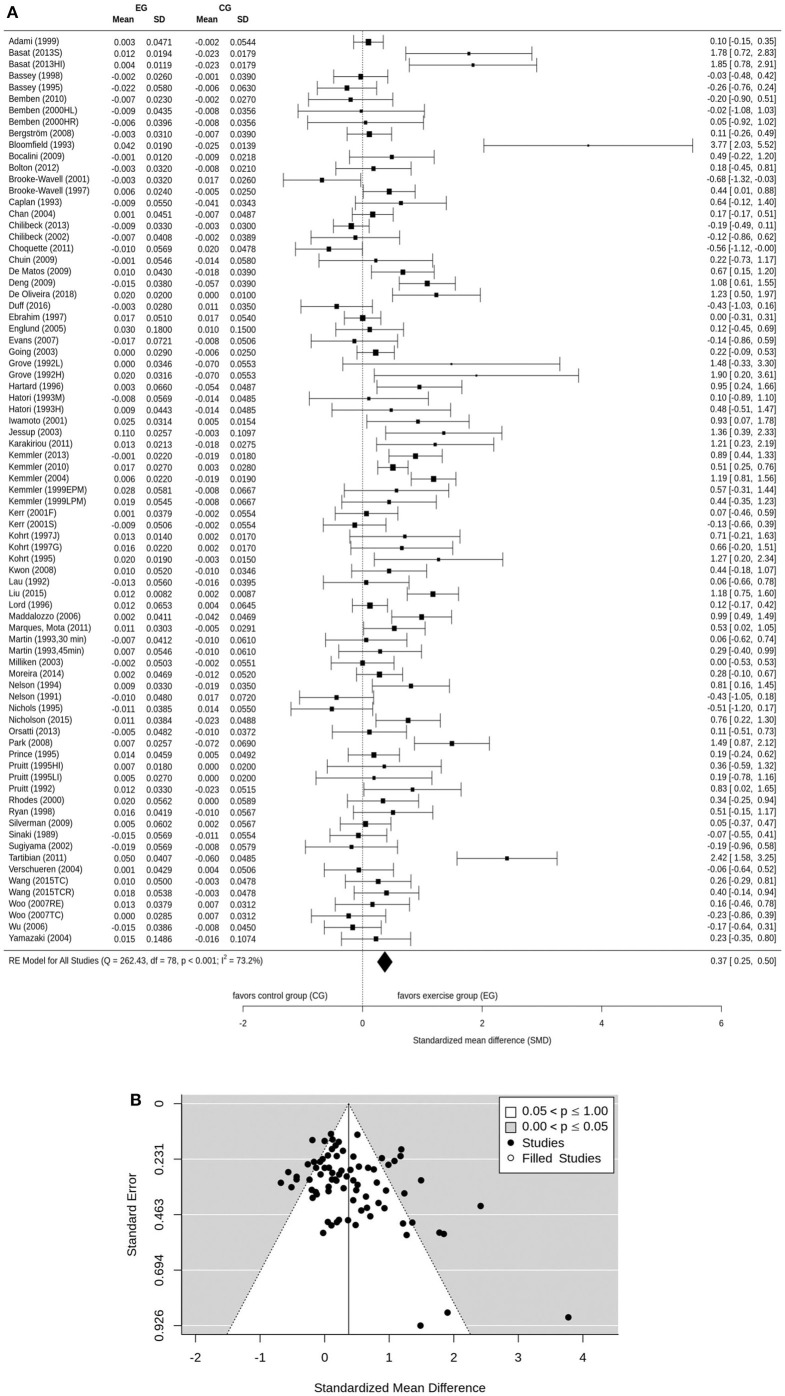
**(A)** Forest plot of meta-analysis results at the LS. The data are shown as pooled standard mean difference (SMD) with 95% CI for changes in exercise and control groups. **(B)** Funnel plot of LS BMD with Trim and Fill. SE, standard error of standardized mean difference; SMD, standardized mean difference.

Seventy-nine trials evaluated the effect of exercise on BMD at the LS. In summary, the exercise intervention resulted in significant positive effects (*P* < 0.001). The pooled estimate of random effect analysis was 0.37, 95%-CI: 0.25–0.50 with a substantial level of heterogeneity between trials [*I*^2^ = 73.2%, Q = 262.43, degrees of freedom (df) = 78, *P* < 0.001; Figure 2A]. Sensitivity analysis revealed the most similar effect, when the mean correlation coefficient (max correlation: SMD = 0.65, 95%-CI: 0.43–0.86; min correlation: SMD = 0.26, 95%-CI: 0.17–0.36) was utilized to impute SD of the absolute change for those studies with missing SDs, and when the analysis was computed among studies with available SDs of the change (25 groups) (SMD = 0.32, 95%-CI: 0.10–0.53, *P* = 0.004). The funnel plot suggested positive evidence of publication bias (Figure 2B). The rank correlation test for funnel plot asymmetry further confirmed the significant asymmetry (*P* = 0.002).

#### Effect of Exercise on BMD at the FN-ROI

**Figure 3:**
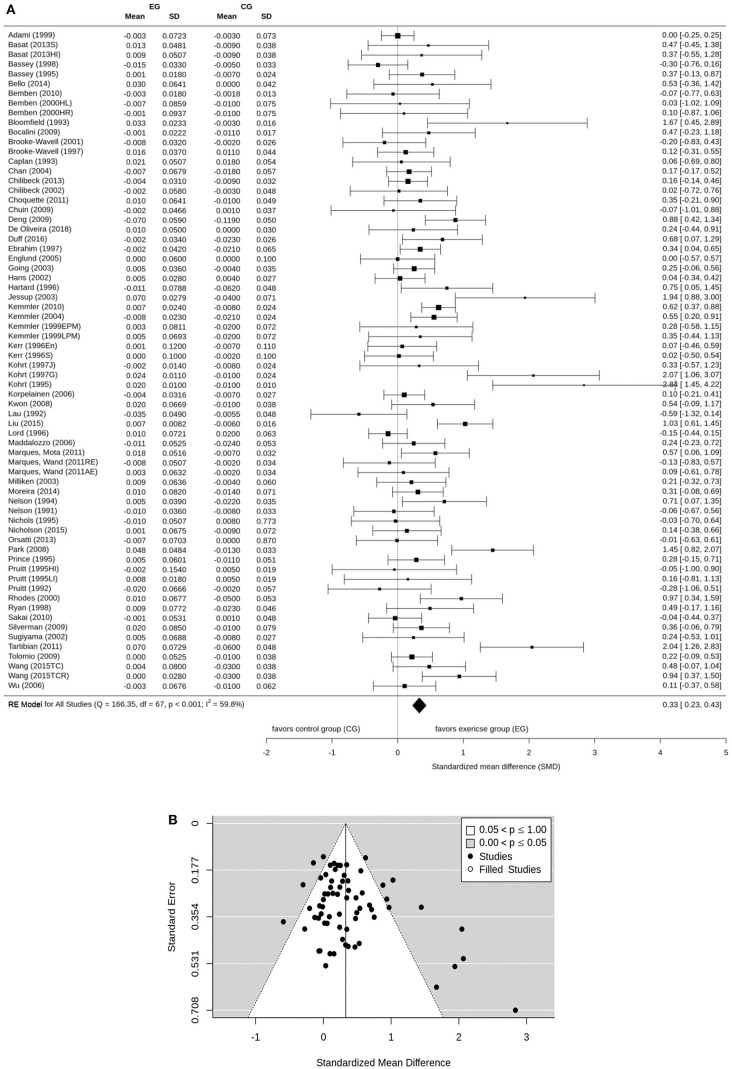
**(A)** Forest plot of meta-analysis results at the FN. The data are shown as pooled standard mean difference (SMD) with 95% CI for changes in exercise and control groups. **(B)** Funnel plot of FN BMD with Trim and Fill. SE, standard error of standardized mean diffterence; SMD, standardized mean difference.

Sixty-eight intervention groups evaluated the effect of exercise on BMD of the FN. The random-effect analysis demonstrated a significant pooled difference between the exercise and control groups (*P* < 0.0001). The pooled estimate of random effect analysis was 0.33, 95%-CI: 0.23–0.43. There was a moderate level of heterogeneity in estimates of the exercise effect [*I*^2^ = 59.8%, Q = 166.35, degrees of freedom (df) = 67, *P* < 0.001; Figure 3A]. Sensitivity analysis indicated the most similar effect when the mean correlation coefficient (max correlation: SMD = 0.74, 95%-CI: 0.49–1.00; min correlation: SMD = 0.24, 95%-CI: 0.16–0.32) was used to impute SD of the absolute change for those trials with missing SDs, and when the analysis was conducted among studies with available SDs of the change (25 groups) (SMD = 0.36, 95%-CI: 0.19–0.52, *P* = 0.0001). The funnel plot suggested positive evidence of publication bias (Figure 3B). The regression test for funnel plot asymmetry presented the significant asymmetry (*P* = 0.03).

#### Effect of Exercise on BMD of Total Hip-ROI

**Figure 4:**
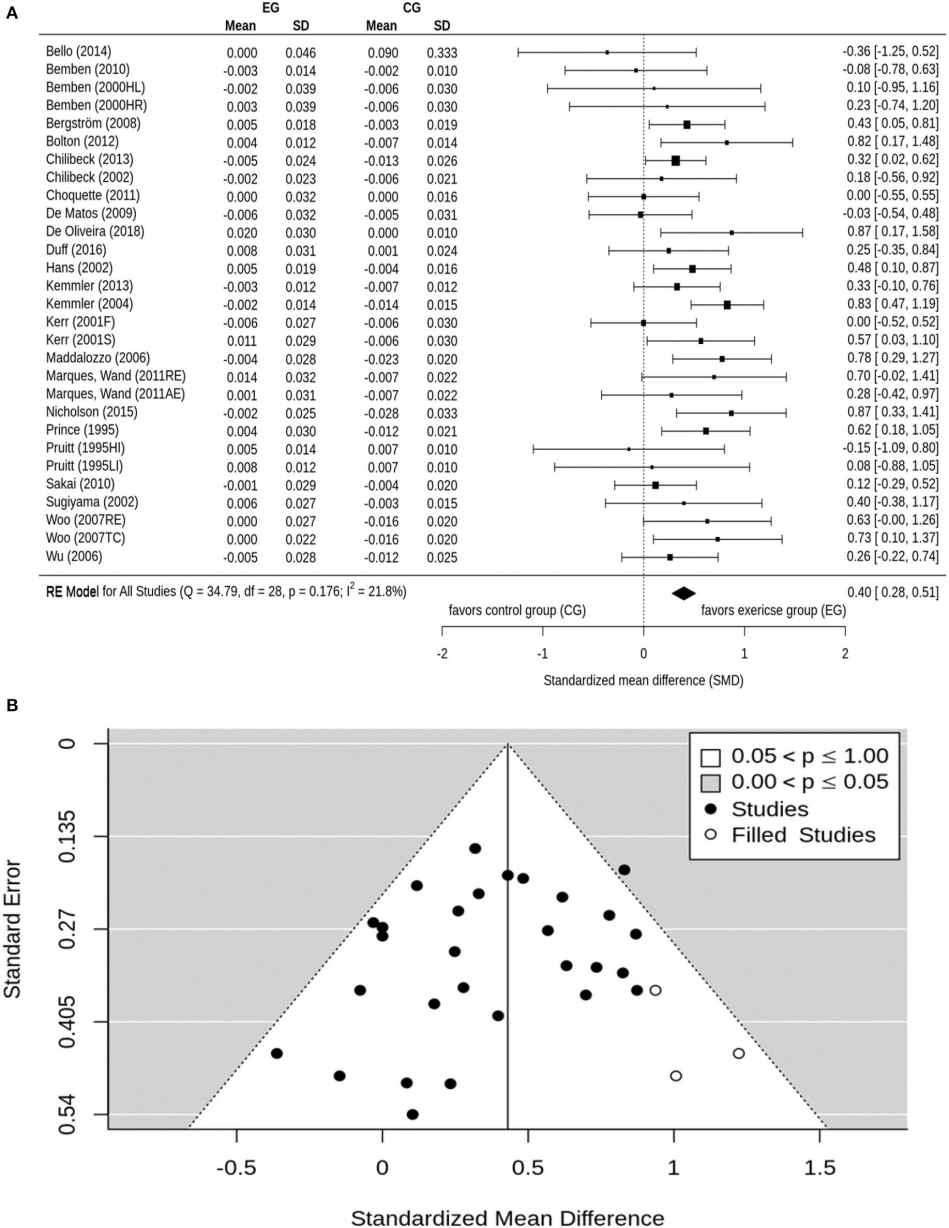
**(A)** Forest plot of meta-analysis results at the total hip. The data are shown as pooled standard mean difference (SMD) with 95% CI for changes in exercise and control groups. **(B)** Funnel plot of total hip BMD with Trim and Fill. SE, standard error of standardized mean difference; SMD, standardized mean difference.

Twenty-nine intervention groups addressed the effect of exercise on BMD of the total Hip. Our result demonstrated a significant exercise-induced improvement in total Hip BMD (*P* < 0.0001). The pooled estimate of random effect analysis, favoring exercise intervention over the control group, was 0.40, 95%-CI: 0.28–0.51. There was a low level of heterogeneity in estimates of the exercise effect [*I*^2^ = 21.8%, Q = 34.79, degrees of freedom (df) = 28, *P* = 0.176; Figure 4A). Sensitivity analysis revealed the most similar effect when the mean correlation coefficient (max correlation: SMD = 0.51, 95%-CI: 0.36–0.66; min correlation: SMD = 0.32, 95%-CI: 0.21–0.42) was used to impute SD of the absolute change for those studies with missing SDs, and when the analysis was computed among studies with available SDs of the change (11 groups) (SMD = 0.39, 95%-CI: 0.19–0.58, *P* < 0.0001). The funnel plot provided no evidence of publication bias (Figure 4B) which was confirmed by the rank correlation test for funnel plot asymmetry (*P* = 0.42).

### Subgroup Analysis

#### Menopausal Status

**LS-BMD:** To estimate the effect of menopausal status on LS BMD, we only included studies that listed information concerning the menopausal status (early vs. late) of their cohorts. In summary, forty-nine groups were analyzed and a mixed-effects analysis found no significant difference between the early (≤ 8 years, 14 groups) and late (> 8 years, 35 groups) (*P* = 0.24) post-menopausal groups. A subgroup analysis that compared the early (Grove and Londeree, 1992; Pruitt et al., 1992; Kemmler, 1999; Bemben et al., 2000; Sugiyama et al., 2002; Chan et al., 2004; Kemmler et al., 2004, 2013; Wu et al., 2006; Maddalozzo et al., 2007; Deng, 2009; Karakiriou et al., 2012) and late-post-menopausal (Nelson et al., 1991; Lau et al., 1992; Bloomfield et al., 1993; Caplan et al., 1993; Kohrt et al., 1995, 1997; Nichols et al., 1995; Prince et al., 1995; Pruitt et al., 1995; Lord et al., 1996; Brooke-Wavell et al., 1997, 2001; Adami et al., 1999; Kemmler, 1999; Rhodes et al., 2000; Iwamoto et al., 2001; Jessup et al., 2003; Verschueren et al., 2004; Yamazaki et al., 2004; Englund et al., 2005; Woo et al., 2007; Kwon et al., 2008; Park et al., 2008; Bocalini et al., 2009; Chuin et al., 2009; de Matos et al., 2009; Bemben et al., 2010; Kemmler et al., 2010; Marques et al., 2011b; Tartibian et al., 2011; Nicholson et al., 2015; Duff et al., 2016) group with their corresponding control-groups indicate comparable effects on LS-BMD (early: SMD = 0.64, 95%-CI: 0.33–0.95 vs. late post-menopausal: 0.39, 0.19–0.59).

**FN-BMD:** Of 68 groups that addressed FN-BMD, 44 exercise groups comprised early or late post-menopausal participants. A mixed-effects analysis found no significant difference between early (≤ 8 years, 10 groups) and late (>8 years, 34 groups) (*P* = 0.65) PMW. The subgroup analysis that compared the early (Pruitt et al., 1992; Kemmler, 1999; Bemben et al., 2000; Sugiyama et al., 2002; Chan et al., 2004; Kemmler et al., 2004; Wu et al., 2006; Maddalozzo et al., 2007; Deng, 2009) vs. the late-post-menopausal exercise-groups (Nelson et al., 1991; Lau et al., 1992; Bloomfield et al., 1993; Caplan et al., 1993; Kohrt et al., 1995, 1997; Nichols et al., 1995; Prince et al., 1995; Pruitt et al., 1995; Lord et al., 1996; Brooke-Wavell et al., 1997, 2001; Adami et al., 1999; Kemmler, 1999; Rhodes et al., 2000; Hans et al., 2002; Jessup et al., 2003; Englund et al., 2005; Korpelainen et al., 2006; Kwon et al., 2008; Park et al., 2008; Bocalini et al., 2009; Chuin et al., 2009; Bemben et al., 2010; Kemmler et al., 2010; Sakai et al., 2010; Marques et al., 2011b,c; Tartibian et al., 2011; Nicholson et al., 2015; Duff et al., 2016) with their corresponding control-groups did not detect different effects of menopausal status on FN-BMD (early: SMD = 0.31; 95%-CI: 0.09–0.52 vs. late-post-menopausal: 0.39, 0.17–0.60).

**Total Hip-BMD:** Twenty studies with tHip-BMD assessment reported the menopausal status of their cohorts. A mixed-effects analysis indicated no statistically significant difference between the early (≤ 8 years, 7 groups) and late (> 8 years, 13 groups) post-menopausal group (*P* = 0.37).

The sub-group analysis did not indicate a different effect of varying menopausal status on BMD at the tHip-ROI [early- (Bemben et al., 2000; Sugiyama et al., 2002; Kemmler et al., 2004, 2013; Wu et al., 2006; Maddalozzo et al., 2007): SMD = 0.51, 95%-CI: 0.27–0.75 vs. late post-menopausal (Prince et al., 1995; Pruitt et al., 1995; Hans et al., 2002; Woo et al., 2007; de Matos et al., 2009; Bemben et al., 2010; Sakai et al., 2010; Marques et al., 2011c; Nicholson et al., 2015; Duff et al., 2016): 0.38, 0.20–0.56].

#### Intervention Duration

**LS-BMD:** Of 79 groups, 25 training groups were included in the short-term intervention (≤ 8 months) group (Bloomfield et al., 1993; Hatori et al., 1993; Hartard et al., 1996; Ryan et al., 1998; Adami et al., 1999; Bemben et al., 2000, 2010; Sugiyama et al., 2002; Jessup et al., 2003; Verschueren et al., 2004; Kwon et al., 2008; Bocalini et al., 2009; Chuin et al., 2009; Silverman et al., 2009; Choquette et al., 2011; Marques et al., 2011b; Tartibian et al., 2011; Karakiriou et al., 2012; Basat et al., 2013; Moreira et al., 2014; Nicholson et al., 2015; de Oliveira et al., 2019), 44 groups were classified as applying a moderate duration (9–18 months) intervention (Nelson et al., 1991, 1994; Grove and Londeree, 1992; Lau et al., 1992; Pruitt et al., 1992, 1995; Martin and Notelovitz, 1993; Bassey and Ramsdale, 1995; Kohrt et al., 1995, 1997; Nichols et al., 1995; Lord et al., 1996; Brooke-Wavell et al., 1997, 2001; Bassey et al., 1998; Kemmler, 1999; Rhodes et al., 2000; Chilibeck et al., 2002; Going et al., 2003; Milliken et al., 2003; Chan et al., 2004; Yamazaki et al., 2004; Englund et al., 2005; Wu et al., 2006; Evans et al., 2007; Maddalozzo et al., 2007; Woo et al., 2007; Bergstrom et al., 2008; Park et al., 2008; de Matos et al., 2009; Deng, 2009; Bolton et al., 2012; Kemmler et al., 2013; Orsatti et al., 2013; Liu et al., 2015; Wang et al., 2015; Duff et al., 2016), and 10 training groups applied a long intervention (≥18 months) (Sinaki et al., 1989; Caplan et al., 1993; Prince et al., 1995; Ebrahim et al., 1997; Iwamoto et al., 2001; Kerr et al., 2001; Kemmler et al., 2004, 2010; Chilibeck et al., 2013). According to a mixed-effects analysis, no significant difference was observed between the sub-groups (*P* = 0.26). However, the short intervention period demonstrated a slightly higher effect (exercise vs. control, SMD = 0.59, 95%-CI: 0.29–0.9) than the moderate (0.30, 0.15–0.45) or the long intervention duration (0.28, −0.15–0.58) that did not significantly differ from control (*P* = 0.06).

**FN-BMD:** Of 68 groups, 25 studies applied a short (Bloomfield et al., 1993; Hartard et al., 1996; Ryan et al., 1998; Bemben et al., 2000, 2010; Sugiyama et al., 2002; Jessup et al., 2003; Kwon et al., 2008; Bocalini et al., 2009; Chuin et al., 2009; Silverman et al., 2009; Sakai et al., 2010; Choquette et al., 2011; Marques et al., 2011b,c; Tartibian et al., 2011; Basat et al., 2013; Bello et al., 2014; Moreira et al., 2014; Nicholson et al., 2015; de Oliveira et al., 2019), 35 groups scheduled a moderate (Nelson et al., 1991, 1994; Lau et al., 1992; Pruitt et al., 1992, 1995; Bassey and Ramsdale, 1995; Kohrt et al., 1995, 1997; Nichols et al., 1995; Kerr et al., 1996; Lord et al., 1996; Brooke-Wavell et al., 1997, 2001; Bassey et al., 1998; Kemmler, 1999; Rhodes et al., 2000; Chilibeck et al., 2002; Going et al., 2003; Milliken et al., 2003; Chan et al., 2004; Englund et al., 2005; Wu et al., 2006; Maddalozzo et al., 2007; Park et al., 2008; Deng, 2009; Tolomio et al., 2009; Orsatti et al., 2013; Liu et al., 2015; Wang et al., 2015; Duff et al., 2016), and 8 groups conducted a long duration of the exercise intervention (Caplan et al., 1993; Prince et al., 1995; Ebrahim et al., 1997; Hans et al., 2002; Kemmler et al., 2004, 2010; Korpelainen et al., 2006; Chilibeck et al., 2013). A mixed-effects analysis did not observe significant differences between the sub-groups (*P* = 0.83). The subgroups analysis demonstrated that the short intervention period triggered the highest effects (exercise vs. control, SMD = 0.38, 95%-CI: 0.20–0.56) followed by moderate (0.32, 0.15–0.49), and long intervention duration (0.30, 0.13–0.47).

**Total Hip-BMD:** Of 29 groups, 11 training groups were classified as short-term (Bemben et al., 2000, 2010; Sugiyama et al., 2002; Sakai et al., 2010; Choquette et al., 2011; Marques et al., 2011c; Bello et al., 2014; Nicholson et al., 2015; de Oliveira et al., 2019), 12 groups were classified as moderate (Pruitt et al., 1995; Chilibeck et al., 2002; Wu et al., 2006; Maddalozzo et al., 2007; Woo et al., 2007; Bergstrom et al., 2008; de Matos et al., 2009; Bolton et al., 2012; Kemmler et al., 2013; Duff et al., 2016), and six training groups were categorized as long-term interventions (Prince et al., 1995; Kerr et al., 2001; Hans et al., 2002; Kemmler et al., 2004; Chilibeck et al., 2013). A mixed-effects analysis indicated no significant difference between the subgroups (*P* = 0.50). In contrast to LS and FN, the subgroup analysis indicated that long-term intervention demonstrated a tendentially more favorable effect on tHip-BMD (exercise vs. control, SMD = 0.48, 95%-CI: 0.27–0.7) than moderate (0.39, 0.23–0.55) or short intervention duration (0.31, 0.06–0.55).

#### Type of Exercise

**LS-BMD:** Of 79 groups, 18 training groups were classified as WB-AE (Nelson et al., 1991; Lau et al., 1992; Hatori et al., 1993; Martin and Notelovitz, 1993; Kohrt et al., 1995, 1997; Prince et al., 1995; Brooke-Wavell et al., 1997, 2001; Ebrahim et al., 1997; Ryan et al., 1998; Yamazaki et al., 2004; Wu et al., 2006; Evans et al., 2007; Silverman et al., 2009; Tartibian et al., 2011), 15 as DRT (Pruitt et al., 1992, 1995; Nelson et al., 1994; Hartard et al., 1996; Bemben et al., 2000; Chilibeck et al., 2002; Maddalozzo et al., 2007; Woo et al., 2007; Orsatti et al., 2013; Nicholson et al., 2015; Duff et al., 2016; de Oliveira et al., 2019), 11 as Jumping+RT+WB (Grove and Londeree, 1992; Bassey and Ramsdale, 1995; Kemmler, 1999; Milliken et al., 2003; Kemmler et al., 2004, 2013; Deng, 2009; Bolton et al., 2012; Karakiriou et al., 2012; Basat et al., 2013), 24 as WB+RT (Grove and Londeree, 1992; Caplan et al., 1993; Nichols et al., 1995; Lord et al., 1996; Adami et al., 1999; Iwamoto et al., 2001; Kerr et al., 2001; Going et al., 2003; Jessup et al., 2003; Verschueren et al., 2004; Englund et al., 2005; Bergstrom et al., 2008; Kwon et al., 2008; Park et al., 2008; Bocalini et al., 2009; Chuin et al., 2009; de Matos et al., 2009; Bemben et al., 2010; Kemmler et al., 2010; Choquette et al., 2011; Marques et al., 2011b; Basat et al., 2013; Chilibeck et al., 2013), two groups as jumping (Bassey et al., 1998; Sugiyama et al., 2002), 4 groups as non-WB+RT (Bloomfield et al., 1993; Kohrt et al., 1997; Rhodes et al., 2000; Moreira et al., 2014), and five training groups as Tai Chi intervention (Chan et al., 2004; Woo et al., 2007; Liu et al., 2015; Wang et al., 2015). A mixed-effects analysis did not reveal significant differences between the subgroups (*P* = 0.36). According to the subgroup analysis, Jumping+RT+WB triggered the most favorable (and reliable) effects on LS-BMD (exercise vs. control, SMD = 0.71, 95%-CI: 0.33–1.10), followed by dynamic RT (0.40, 0.13–0.67) and the WB+RT intervention (0.30, 0.10–0.50). There was a considerable variation of study effects in the WB-AE (18 groups, 0.24, −0.03 −0.52), Tai Chi (5 groups, 0.37, −0.08 to 0.83), Non-WB+RT (4 groups, 1.05, −0.31 to 2.50) -groups with no significant differences to control in the three latter groups. Of note, the (two) jumping only studies revealed a slight trend to negative effects on BMD (−0.07, −0.46 to 0.32).

**FN-BMD:** Of 68 training groups, 15 were classified as WB-AE (Nelson et al., 1991; Lau et al., 1992; Kohrt et al., 1995, 1997; Prince et al., 1995; Brooke-Wavell et al., 1997, 2001; Ebrahim et al., 1997; Ryan et al., 1998; Hans et al., 2002; Wu et al., 2006; Silverman et al., 2009; Sakai et al., 2010; Marques et al., 2011c; Tartibian et al., 2011), 15 as DRT (Pruitt et al., 1992, 1995; Nelson et al., 1994; Hartard et al., 1996; Kerr et al., 1996; Bemben et al., 2000; Chilibeck et al., 2002; Maddalozzo et al., 2007; Orsatti et al., 2013; Nicholson et al., 2015; Duff et al., 2016; de Oliveira et al., 2019), 8 as Jumping+RT+WB (Bassey and Ramsdale, 1995; Kemmler, 1999; Milliken et al., 2003; Kemmler et al., 2004; Korpelainen et al., 2006; Deng, 2009; Basat et al., 2013), 20 as WB+RT (Caplan et al., 1993; Nichols et al., 1995; Lord et al., 1996; Adami et al., 1999; Going et al., 2003; Jessup et al., 2003; Englund et al., 2005; Kwon et al., 2008; Park et al., 2008; Bocalini et al., 2009; Chuin et al., 2009; Tolomio et al., 2009; Bemben et al., 2010; Kemmler et al., 2010; Choquette et al., 2011; Marques et al., 2011b,c; Basat et al., 2013; Chilibeck et al., 2013; Bello et al., 2014), 2 as jumping (Bassey and Ramsdale, 1995; Sugiyama et al., 2002), 4 as non-WB+RT (Bloomfield et al., 1993; Kohrt et al., 1997; Rhodes et al., 2000; Moreira et al., 2014), and 4 as Tai Chi exercise type (Chan et al., 2004; Liu et al., 2015; Wang et al., 2015). A mixed-effects analysis did not result in significant differences between the subgroups (*P* = 0.43). According to the subgroup analysis, the Non-WB+RT (4 groups, SMD = 0.68, 95%-CI: 0.16–1.19) and the Tai Chi (4 groups, 0.64, 0.21–1.05) demonstrated the most favorable effects (vs. corresponding control), followed by WB-AE (0.42, 0.03–0.81), Jumping+RT+WB (0.39, 0.17–0.62), WB+RT (0.30, 0.12–0.48) and DRT (0.21, 0.04–0.38). A tangentially negative effect was observed for the Jumping subgroup (2 studies, −0.12, −0.62 to 0.37).

**Total Hip-BMD:** Of 29 groups, five training groups were considered as WB-AE (Prince et al., 1995; Hans et al., 2002; Wu et al., 2006; Sakai et al., 2010; Marques et al., 2011c), 10 groups as DRT (Prince et al., 1995; Bemben et al., 2000; Chilibeck et al., 2002; Maddalozzo et al., 2007; Woo et al., 2007; Nicholson et al., 2015; Duff et al., 2016; de Oliveira et al., 2019), three groups as Jumping+RT+WB (Kemmler et al., 2004, 2013; Bolton et al., 2012), and 9 groups as WB+RT (Kerr et al., 2001; Bergstrom et al., 2008; de Matos et al., 2009; Bemben et al., 2010; Choquette et al., 2011; Marques et al., 2011c; Chilibeck et al., 2013; Bello et al., 2014). The Jumping (Sugiyama et al., 2002) and Tai Chi (Woo et al., 2007) groups comprised only one intervention group, thus they were excluded from the analysis. Based on the mixed-effects analysis, no significant differences were seen between the subgroups (*P* = 0.08). According to the subgroup analysis, Jumping+RT+WB showed the largest effect (exercise vs. control, SMD = 0.65, 95%-CI: 0.30–1.00) followed by the DRT (0.51, 0.28–0.74), the WB-AE (0.36, 0.16–0.56), and the WB+RT group (0.24, 0.08–0.41).

#### Ground-Reaction Forces (GRF) and Joint-Reaction Forces (JRF)

Finally, study interventions were categorized in GRF, JRF or mixed (GRF and JRF) mechanical forces.

**LS-BMD:** Of 79 groups, 19 training groups applied JRF exercise (Sinaki et al., 1989; Pruitt et al., 1992, 1995; Bloomfield et al., 1993; Nelson et al., 1994; Hartard et al., 1996; Kohrt et al., 1997; Bemben et al., 2000; Rhodes et al., 2000; Chilibeck et al., 2002; Maddalozzo et al., 2007; Woo et al., 2007; Orsatti et al., 2013; Moreira et al., 2014; Nicholson et al., 2015; Duff et al., 2016; de Oliveira et al., 2019), 20 applied GRF exercise (Nelson et al., 1991; Lau et al., 1992; Hatori et al., 1993; Martin and Notelovitz, 1993; Bassey and Ramsdale, 1995; Kohrt et al., 1995, 1997; Prince et al., 1995; Brooke-Wavell et al., 1997, 2001; Ebrahim et al., 1997; Bassey et al., 1998; Sugiyama et al., 2002; Yamazaki et al., 2004; Wu et al., 2006; Silverman et al., 2009; Tartibian et al., 2011; Basat et al., 2013), and 35 studies prescribed mixed mechanical forces protocols (Grove and Londeree, 1992; Caplan et al., 1993; Nichols et al., 1995; Lord et al., 1996; Ryan et al., 1998; Adami et al., 1999; Kemmler, 1999; Iwamoto et al., 2001; Kerr et al., 2001; Going et al., 2003; Jessup et al., 2003; Milliken et al., 2003; Kemmler et al., 2004, 2010, 2013; Verschueren et al., 2004; Englund et al., 2005; Evans et al., 2007; Bergstrom et al., 2008; Kwon et al., 2008; Park et al., 2008; Bocalini et al., 2009; Chuin et al., 2009; de Matos et al., 2009; Deng, 2009; Bemben et al., 2010; Choquette et al., 2011; Marques et al., 2011b; Bolton et al., 2012; Karakiriou et al., 2012; Basat et al., 2013; Chilibeck et al., 2013). A further of 5 training groups (Chan et al., 2004; Woo et al., 2007; Liu et al., 2015; Wang et al., 2015), could not be reliably classified within one of the categories therefore we excluded them from the subgroup analysis. A mixed-effects analysis found no significant differences between the categories (*P* = 0.46). According to the subgroup analysis, JRF exercise triggered the highest effect on LS-BMD (exercise vs. control, SMD = 0.46, 95%-CI: 0.21–0.70), followed by the mixed JRF and GRF (0.41, 0.22–0.59). GRF exercise however, did not significantly (*P* = 0.09) differ from corresponding control (0.24, −0.04 to 0.53).

**FN-BMD:** Of 78 groups, 19 training groups were classified as JRF type exercise (Pruitt et al., 1992; Bloomfield et al., 1993; Nelson et al., 1994; Prince et al., 1995; Hartard et al., 1996; Kerr et al., 1996; Kohrt et al., 1997; Bemben et al., 2000; Rhodes et al., 2000; Chilibeck et al., 2002; Maddalozzo et al., 2007; Orsatti et al., 2013; Moreira et al., 2014; Nicholson et al., 2015; Duff et al., 2016; de Oliveira et al., 2019), 18 as GRF (Nelson et al., 1991; Lau et al., 1992; Bassey and Ramsdale, 1995; Kohrt et al., 1995, 1997; Prince et al., 1995; Brooke-Wavell et al., 1997, 2001; Ebrahim et al., 1997; Bassey et al., 1998; Hans et al., 2002; Sugiyama et al., 2002; Korpelainen et al., 2006; Wu et al., 2006; Silverman et al., 2009; Marques et al., 2011c; Tartibian et al., 2011; Basat et al., 2013) and 26 groups as mixed JRF and GRF protocols (Caplan et al., 1993; Nichols et al., 1995; Lord et al., 1996; Ryan et al., 1998; Adami et al., 1999; Kemmler, 1999; Going et al., 2003; Jessup et al., 2003; Milliken et al., 2003; Kemmler et al., 2004, 2010; Englund et al., 2005; Kwon et al., 2008; Park et al., 2008; Bocalini et al., 2009; Chuin et al., 2009; Deng, 2009; Tolomio et al., 2009; Bemben et al., 2010; Choquette et al., 2011; Marques et al., 2011b,c; Basat et al., 2013; Chilibeck et al., 2013; Bello et al., 2014). Five training groups cannot be reliably classified (Chan et al., 2004; Sakai et al., 2010; Liu et al., 2015; Wang et al., 2015), therefore they were excluded from the sub-group analysis. A mixed-effects analysis demonstrated no significant differences between the subgroups (*P* = 0.89). All the groups demonstrated comparable significant effects on FN-BMD (JRF: SMD = 0.29, 95%-CI: 0.14–0.44 vs. GRF: 0.35, 0.03–0.66 vs. JRF and GRF: 0.34, 0.19–0.49).

**Total Hip-BMD:** Of 29 groups, 10 training groups were included in the JRF group (Pruitt et al., 1995; Bemben et al., 2000; Chilibeck et al., 2002; Maddalozzo et al., 2007; Woo et al., 2007; Nicholson et al., 2015; Duff et al., 2016; de Oliveira et al., 2019). Five intervention groups were classified as GRF (Prince et al., 1995; Hans et al., 2002; Sugiyama et al., 2002; Wu et al., 2006; Marques et al., 2011c) and 12 groups as mixed intervention (Kerr et al., 2001; Kemmler et al., 2004, 2013; Bergstrom et al., 2008; de Matos et al., 2009; Bemben et al., 2010; Choquette et al., 2011; Marques et al., 2011c; Bolton et al., 2012; Chilibeck et al., 2013; Bello et al., 2014). Two training groups (Woo et al., 2007; Sakai et al., 2010) that could not be reliably classified were excluded. A mixed-effects analysis found no significant differences between the subgroups (*P* = 0.57). According to the subgroup analysis, effect size in the JRF-group was largest (SMD = 0.51, 95%-CI: 0.28–0.74), followed by the GRF (0.44, 0.22–0.66) and the mixed JRF and GRF subgroup (0.34, 0.14–0.53) obtained a positive significant difference in comparison with control groups.

## Discussion

A considerable number of systematic reviews and meta-analyses focus on the effect of exercise on BMD at the LS and/or proximal femur. With few exceptions (for LS; Howe et al., 2011) most studies reported low effect sizes (SMD = 0.2–0.5) on average (e.g., Kelley, 1998a,b; Martyn-St. James and Caroll, 2006; Howe et al., 2011; Marques et al., 2011a; Zhao et al., 2017). Due to continued research in the area, we have been able to include more exercise studies in our analysis than previous works (e.g., Howe et al., 2011; Marques et al., 2011a; Zhao et al., 2017). Nevertheless, our finding (SMD-LS = 0.37, SMD-FN = 0.33, SMD-tHip = 0.40) confirmed the results of a significant, but rather small effect of exercise on BMD, at the LS or a relevant proximal femur-ROIs. We largely attribute this finding of limited increase in BMD to the widely diverging effect sizes (e.g., Figures 2A, 3B) across the exercise trials included. Apart from participants' characteristics, considerable differences in exercise characteristics might explain these striking variations among the included trials. We sought to identify parameters that affect the impact of exercise on BMD. Therefore, studies were classified according to (1) menopausal status (Kemmler, 1999; Beck and Snow, 2003), (2) type of exercise (Giangregorio et al., 2014; Beck et al., 2017; Daly et al., 2019), (3) type of mechanical forces (JRF, GRF, JRF and GRF) (Martyn-St James and Carroll, 2011; Daly et al., 2019), and (4) duration of the intervention. Menopausal status might be an important predictor of exercise effects on BMD (Kemmler, 1999), due to the high bone-turnover during the early-menopausal years (Tella and Gallagher, 2014). However, the corresponding subgroup analysis did not determine significant differences or a consistent trend for all BMD-regions (LS, FN, tHip). Type of exercise and mechanical forces were included since mechanistically, they might be the most crucial predictors for the effect of exercise on bone (Giangregorio et al., 2014; Beck et al., 2017; Daly et al., 2019), while longer exposure to exercise (i.e., intervention duration) should result in higher effects on bone, at least when strain was regularly adjusted (“progression”) (Kemmler et al., 2015). Accepting the viewpoint that exercise-induced BMD changes were predominately generated by remodeling (Eriksen, 2010), and considering the length of a remodeling cycle in (older) adults (Eriksen, 2010; Bonucci and Ballanti, 2014), interventions ≤ 8 months might be too short to determine the full extent of bones mineralization^1^. In contrast, although non-significant, the subgroup analysis demonstrated considerably higher effects on LS-BMD among studies with short compared with moderate or long durations (SMD = 0.59 vs. 0.30 vs. 0.28). Based on bone physiology (Eriksen, 2010), it is rather unlikely that exercise interventions ≤ 8 months resulted in higher increases in BMD-LS compared with interventions 18 months and longer. We attribute this dubious finding to the complex interaction of exercise parameters that might have confounded the interaction between training frequency and BMD-LS.

Significant differences in BMD changes within the corresponding subgroups was not detected. Tendentially negative effects of jumping exercise on LS- and FN-BMD^2^ or the trend (*p* = 0.06) to higher effects of short exercise duration on LS and FN-BMD was observed.

We did not address exercise intensity (Rubin and Lanyon, 1985; Frost, 2003) or -frequency (Kemmler and von Stengel, 2013; Kemmler et al., 2016), which is a key modulator of effective exercise protocols (Weineck, 2019). It was planned to include “exercise intensity” in the subgroups analysis; however, it was not possible to present a meaningful and comprehensive rating of all the studies^3^. Since 15 studies did not report attendance rate and therefore the factual training frequency remained vague, exercise frequency was not evaluated.

Due to the results of the (exercise) group comparisons and subgroup analysis, we are unable to give validated exercise recommendations for optimized bone-strengthening protocols for PMW. In this context, Gentil et al. (2017) questioned whether “there is any practical application of meta-analytic results in strength training.” This might be overstating the issue; however trying to derive exercise recommendations and, to a lesser degree, the proper effect size estimation will fail when addressing varying exercise interventions “en bloc.” Several aspects support this view. First, exercise is a very complex intervention. The type of exercise alone ranges from HIT-RT or depth jumps, for example, to brisk walking, chair exercises and balance training. Additionally, exercise parameters (intensity, duration, cycle number, frequency etc.; Toigo and Boutellier, 2006; Weineck, 2019) and training principles (e.g., progression, periodization etc.; Weineck, 2019), fundamentally modify the effect of the exercise type on a given study endpoint. Even minor variations in single exercise parameters can result in considerable differences in BMD changes (e.g., Kemmler et al., 2016). In parallel, the present analysis indicates that a lack of consistent progression might prevent further BMD changes after initial adaptations^4^, according to non-compliance with the overload principle (Weineck, 2019). At this point, a frequent limitation of exercise research arises: Unlike in pharmaceutical trials, the general effectiveness of the exercise protocol was rarely evaluated before the initiation of the clinical trial (phase III) (Umscheid et al., 2011). Further, in some cases, there is an impression that some older studies (Bloomfield et al., 1993; Brooke-Wavell et al., 1997, 2001) evaluate the least significant effect of exercise on bone. This further contributes to the considerable “apple-oranges problem” (Esteves et al., 2017; Milojevic et al., 2018) of meta-analysis in the area of “exercise.” In summary thus, we conclude that uncritical acceptance of the acquired meta-analytic data (particularly) of exercise studies is certainly unwarranted.

Some study limitations may decrease the validity of our study. The lack of information related to participant and exercise characteristics and in the case of missing responses after contacting the authors meant that we estimated some variables. For example, in studies that did not provide the menopausal status of their participants, we consider the age of 51 years as the menopausal transition age to estimate the post-menopausal age (Palacios et al., 2010). Further, we excluded studies that included participants with pharmaceutic agents or diseases, known to relevantly affect BMD, in order to prevent a confounding, synergistic/additive/permissive effect on our study endpoints. However, due to the lack of information in most individual studies, we were unable to adjust for changes of medication, diet or emerging diseases.

Another predominately biometrical issue was that SDs of the absolute change in BMD were not consistently available and have thus to be imputed, which may have reduced the accuracy of the data. Further, there is considerable evidence for a publication bias with respect to exercise-induced BMD changes at the LS and tHip. Considering the aspect that most authors tend to reported positive effects the true effect size of exercise on BMD might be slightly lower compared to the results presented here (Sterne et al., 2011).

The main limitation was the extensive approach of including all types of exercise in the main analysis, which resulted in large variations in effects sizes. Moreover, our inability to categorize adequately relevant exercise characteristics hinders the proper comparison of homogeneous and widely independent subgroups and thus prevents validated exercise recommendations. Hence, upcoming meta-analysis in the area of exercise on bone should focus on dedicated areas of exercise. However, we conclude that well-designed randomized controlled trials which allow adjusting for one single parameter while keeping all others constant might be the better option for evaluating the contribution of participants and exercise parameters on exercise effect on bone and deriving sophisticated recommendations for exercise.

## Conclusion

In summary, our approach of (1) including heterogeneous exercise studies, (2) categorizing them according to relevant modulators and exercise parameters, and (3) comparing the corresponding subgroups to identify modulators of exercise effects on bone and (more important) the most favorable exercise protocol on bone by means of enhanced statistics ultimately failed. This result can be largely attributed to fundamental and complex differences among the exercise protocols of the large amount of exercise studies included, which in effect prevent a meaningful categorization of exercise parameters.

## Data Availability Statement

The datasets generated during and/or analyzed during the current study are available from the first author (mahdieh.shojaa@imp.uni-erlangen.de) at the Institute of Medical Physics of Friedrich-Alexander University Erlangen-Nürnberg upon reasonable request.

## Author Contributions

MS and WK initiated the Meta-analysis. The literature search was done by MS. MS, SV, MK, DS, and WK performed data analysis, interpretation, and drafted the manuscript. MS, WK, SV, MK, DS, GB, LB, LD, SM, MHM, AS, MM, MJ, and TR contributed to quality assessment and revised the manuscript. WK accepted responsibility for the integrity of the data sampling, analysis and interpretation. All authors contributed to the article and approved the submitted version.

## Conflict of Interest

The authors declare that the research was conducted in the absence of any commercial or financial relationships that could be construed as a potential conflict of interest.

## Footnotes

^1^Taking into account that DXA only determines the mineralized bone matrix (i.e., BMD).

^2^Most recommendations (e.g., Beck et al., 2017; Daly et al., 2019; Kemmler and von Stengel, 2019), however, consider Jumping as a favorable of type of exercise for PMW.

^3^The classification of exercise intensity in the area of bone research is not trivial. WK and SV failed to generate a reliable classification of exercise intensity/strain magnitude across the (endurance and resistance type) studies of the present review.

^4^We speculate that lack of progression contribute to the result of the subgroup analysis that address intervention-duration.
